# Fast, accurate ranking of engineered proteins by target-binding propensity using structure modeling

**DOI:** 10.1016/j.ymthe.2024.04.003

**Published:** 2024-04-06

**Authors:** Xiaozhe Ding, Xinhong Chen, Erin E. Sullivan, Timothy F. Shay, Viviana Gradinaru

**Affiliations:** 1 Division of Biology and Biological Engineering, California Institute of Technology, 1200 E California, Boulevard, Pasadena, CA 91125, USA

**Keywords:** protein engineering, AAV engineering, *in silico* screening, protein binders, protein structure prediction, protein design, binder ranking, AlphaFold, receptor binding, receptor engagement

## Abstract

Deep-learning-based methods for protein structure prediction have achieved unprecedented accuracy, yet their utility in the engineering of protein-based binders remains constrained due to a gap between the ability to predict the structures of candidate proteins and the ability toprioritize proteins by their potential to bind to a target. To bridge this gap, we introduce Automated Pairwise Peptide-Receptor Analysis for Screening Engineered proteins (APPRAISE), a method for predicting the target-binding propensity of engineered proteins. After generating structural models of engineered proteins competing for binding to a target using an established structure prediction tool such as AlphaFold-Multimer or ESMFold, APPRAISE performs a rapid (under 1 CPU second per model) scoring analysis that takes into account biophysical and geometrical constraints. As proof-of-concept cases, we demonstrate that APPRAISE can accurately classify receptor-dependent vs. receptor-independent adeno-associated viral vectors and diverse classes of engineered proteins such as miniproteins targeting the severe acute respiratory syndrome coronavirus 2 (SARS-CoV-2) spike, nanobodies targeting a G-protein-coupled receptor, and peptides that specifically bind to transferrin receptor or programmed death-ligand 1 (PD-L1). APPRAISE is accessible through a web-based notebook interface using Google Colaboratory (https://tiny.cc/APPRAISE). With its accuracy, interpretability, and generalizability, APPRAISE promises to expand the utility of protein structure prediction and accelerate protein engineering for biomedical applications.

## Graphical abstract

**Figure:**
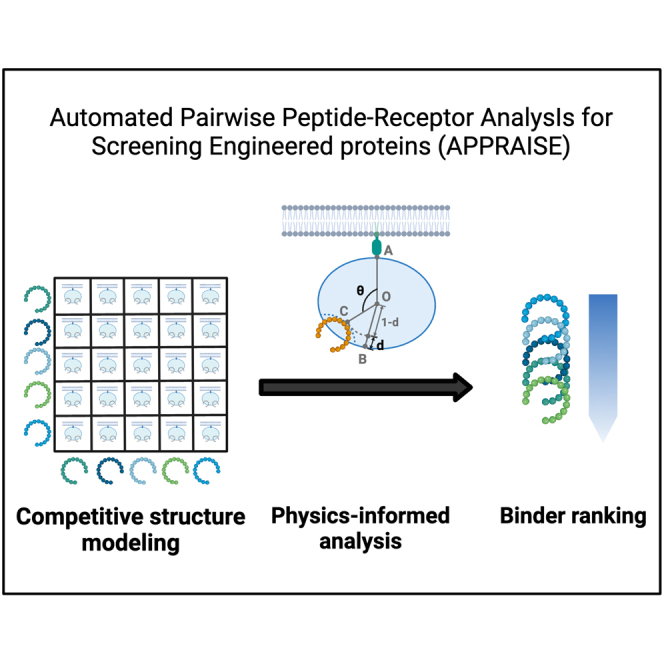

## Teaser

Ding and colleagues address the challenge of predicting engineered proteins' target-binding capabilities by leveraging structure prediction engines such as AlphaFold. Their tool, APPRAISE, combines deep learning with structural insights to rapidly assess many predicted structures, thereby judging engineered proteins’ binding potential and facilitating efficient therapy development.

## Introduction

Many protein-based biologics rely on precise targeting. As a result, protein engineers have devoted considerable effort to create specific binders, using methods such as directed evolution^1^^,^^2^^,^^3^^,^^4^ and rational design.^5^^,^^6^^,^^7^ Currently, the costly experimental evaluation of candidate binders using *in vitro* and *in vivo* assays presents a bottleneck, which can be eased using computational prioritization.^8^

Two strategies are employed to predict protein functions: end-to-end sequence-function and two-step sequence-structure/structure-function. End-to-end sequence-function models can predict complex functions such as enzyme activities or ion channel conductivity,^9^^,^^10^ which are challenging to calculate using physical principles.^11^ However, such specialized models require domain-specific, high-quality training datasets for accurate prediction. In comparison, the two-step sequence-structure/structure-function strategy offers a more generalizable solution, particularly for functions with well-understood biophysical mechanisms such as protein-protein binding.

The rapid development of deep-learning-based methods has brought unprecedented accuracy to the first step of the sequence-structure/structure-function strategy. Since AlphaFold2 (AF2)’s outstanding performance in CASP14 in 2020,^12^ several new deep-learning-based structure prediction tools have been released,^13^^,^^14^^,^^15^^,^^16^^,^^17^^,^^18^^,^^19^^,^^20^^,^^21^^,^^22^^,^^23^^,^^24^ providing a diverse toolset for generating protein models with atomic-level precision. While the original AF2 can predict protein-protein complexes,^25^ there are enhanced versions such as AlphaFold-Multimer that can model multi-chain complexes with greater accuracy.^14^^,^^17^ Importantly, these structure prediction tools allow the generation of models in less than one GPU hour each, a level of throughput that experimental methods cannot match.

The second step, ranking target-binding propensities based on structure predictions, has been less attended to than the first. Structure prediction tools generate confidence scores for predicted multimer models, such as predicted local distance difference test (pLDDT) and predicted template modeling (pTM) scores (used by AF2),^12^ and interface pTM scores (used by AF-Multimer),^14^ which have been used off label as metrics to evaluate the probability of binding.^17^^,^^26^ However, previous reports^27^ and our experience revealed that these scores alone are, in some cases, not reflective of binding propensities, particularly when the interaction is weak or transient. Extracting additional information stored in the 3D coordinates using biophysical principles may help improve the accuracy of binder ranking.

Ranking the binding probability of engineered proteins through modeled structures presents unique challenges. A frequent challenge is imposed by the high sequence similarity between candidate molecules. Engineered protein variants are often constructed by modifying a short variable region in a common scaffold. Due to this similarity, the energy difference between the candidate binders can be very small, sometimes buried in the error of the energy function used for candidate ranking.^28^^,^^29^ This problem is compounded by structure prediction methods that rely heavily on co-evolutionary information or homology, causing them to generate similar binding poses for the candidate proteins. Another major challenge is assessing a large number of predicted structure models efficiently. Direct quantification of protein-protein interface energy using interpretable, physics-based methods trades off between accuracy and speed.^30^ For instance, molecular dynamics simulation methods can cost more than $10^{3}$ CPU hours per model. Faster, less rigorous methods with better-than-random ability to predict the impact of interface mutations still require 1 CPU minute to 1 CPU hour per non-antibody-antigen model.^30^ In the post-AlphaFold era, an interpretable and efficient method of predicting the target binding of a large number of models would greatly accelerate protein engineering efforts.

Recently, Chang and Perez utilized competitive modeling with AF-Multimer to demonstrate a correlation between competition results and peptide-binding affinities.^27^ However, the study’s method of assessing the competition results necessitates a comparison of the modeled structures to an experimentally solved "native" structure, which is not available for many engineered proteins.

To bridge the remaining gap between structure prediction and protein engineering, here we present Automated Pairwise Peptide-Receptor Analysis for Screening Engineered proteins (APPRAISE), a readily interpretable and generalizable method for ranking the target-binding propensity of engineered proteins based on competitive structure modeling and fast physics-informed structure analysis.

## Results

**Figure 1:**
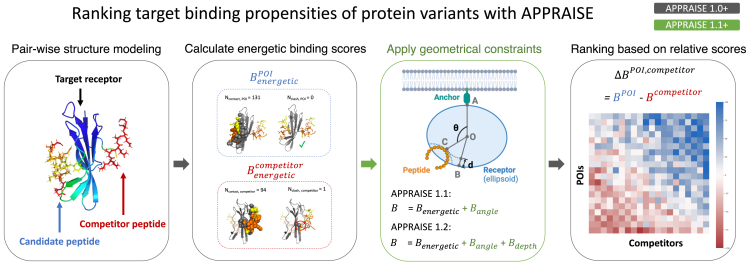
Workflow of APPRAISE First, engineered protein candidates or peptides from the protein candidates’ target-binding region are modeled in competing pairs with the target receptor using tools such as AF-Multimer or ESMFold. Second, a non-negative energetic binding score based on atom counting is calculated for each peptide. Third, in APPRAISE 1.1+, additional geometrical constraints critical for peptide binding, including the binding angle and pocket depth, are considered. Finally, a relative score for each match is calculated by taking the difference between the scores for the two peptides. The averaged relative scores form a matrix that determines the final ranking.

The workflow of APPRAISE (Figure 1) comprises four main components. In the first step, pairs of peptides from N candidate protein molecules ($N^{2}$ pairs total) are modeled in complex with a target receptor using a state-of-the-art structure method such as AF-Multimer.^14^ In the second stage, a simplified energetic binding score is calculated for each peptide (i.e., the peptide of interest [POI] and its competitor). In the third optional step, geometrical constraints for effective binding are applied to these scores. Finally, the result of each competition is decided using the score difference between the POI and the competitor, and the peptides are ranked based on the matrix of competition results.

### APPRAISE can accurately classify receptor-mediated brain transduction of viral vectors

We first developed APPRAISE to predict the binding propensities of engineered adeno-associated virus (AAV) capsids for brain receptors. Recombinant AAVs are widely used as delivery vectors for gene therapy due to their relative safety as well as their broad and engineerable tropism. *In vivo* selections from libraries of randomized peptide-displaying AAV variants have yielded capsids that can transduce the animal brain,^1^^,^^2^^,^^31^^,^^32^^,^^33^^,^^34^^,^^35^ an organ tightly protected by the blood-brain barrier (BBB). Widely known examples among these capsids are AAV-PHP.B^1^ and AAV-PHP.eB,^31^ two AAV9-based^36^ variants displaying short (7–9 amino acids) surface peptides. The two variants can efficiently deliver genetic cargo to the brains of a subset of rodent strains. Genetic and biophysical studies have revealed that the BBB receptor for PHP.B/PHP.eB in these strains is LY6A, a GPI-anchored membrane protein.^37^^,^^38^^,^^39^ A dataset comprising peptide-displaying AAV capsids that were engineered in a similar way as PHP.B/eB was collected in order to train the APPRAISE method (Figure S1). Although binding between the AAV and the LY6A receptor is dynamic^40^^,^^41^ and therefore challenging to quantitatively measure, we could infer the binary LY6A-binding profiles of AAV capsids from their differential brain transduction profiles in mouse strains with and without the receptor, producing a training set of peptide-displaying AAV capsids (Figure S1).

**Figure 2:**
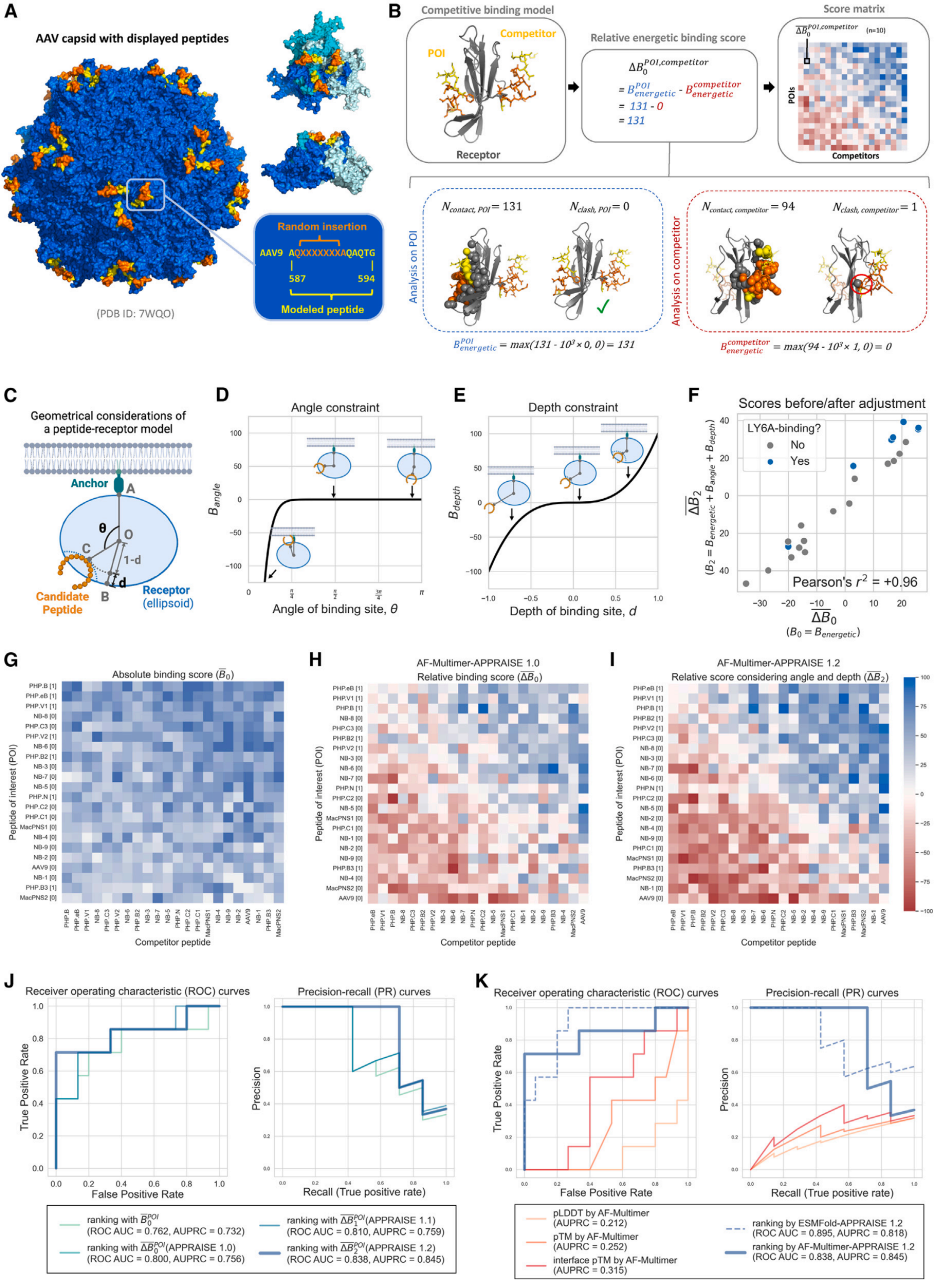
Binary classification of receptor-binding AAV capsids using physical and geometrical principles (A) A structure model of AAV-PHP.eB, highlighting the site for inserting the displayed peptide (orange) and the peptide used for APPRAISE modeling (yellow or orange). The left image shows the AAV capsid of 60 structurally identical subunits. The two images on the top right show a top view and a side view around the 3-fold axis, respectively. The three subunits that make the trimer are colored blue, cyan, and white. The sequence corresponding to the peptides is shown in the bottom right. (B) An example showing the calculation process of a relative energetic binding score. The number of contacting atoms (<5Å) and the number of clashing atoms (<1Å) for each peptide in the competition are counted, and an absolute energetic binding score is calculated based on the counts according to Equation 1. A difference between the two numbers, or the relative energetic binding score, is then calculated. The competition result between two peptides is determined using the average of relative binding scores across replicates. The matrix of the mean scores is then used to rank the peptides of interest (POIs). (C) A simplified geometrical representation of a peptide-receptor model, where the hull of the receptor is represented by an ellipsoid (blue). Point O, the center of mass of the receptor; point A, the receptor’s terminus attached to an anchor; segment OB, the minor axis of the ellipsoid receptor hull; point C, the deepest point on the candidate peptide (orange); θ, the binding angle of the peptide; d, the binding pocket depth of the peptide. (D) The angle constraint function. Three representative scenarios with different binding angles are highlighted. (E) The depth constraint function. Three representative scenarios with different binding depths are highlighted. (F) Comparison of the averaged relative binding energy scores before geometry-based adjustments vs. after adjustments. (G–I) Heatmaps representing the matrix of mean scores of 22 AAV9-based capsid variants, including (G) mean absolute binding scores, (H) mean relative binding scores, and (I) mean relative binding scores that have considered both angle and depth constraints. All heatmap matrices were sorted by point-based round-robin tournaments (section “materials and methods”). Bracketed numbers in the row labels are LY6A-binding profiles of the capsids inferred from experimental evidence (Figure S1). Each block in the heatmap represents the mean score measured from 10 independent models generated by AlphaFold-Multimer. (J–K) Comparison of different ranking methods used as binary classifiers to predict the LY6A-binding profile of 22 AAV9-based capsid variants. (J) Comparison between rankings given by different versions of APPRAISE scores using AF-Multimer as the structure prediction tool. (K) Comparison between rankings given by confidence scores of AF-Multimer versus rankings given by APPRAISE 1.2 using either AF-Multimer or ESMFold as prediction engines. The sequence and shape parameters of LY6A used for the modeling and analyses are included in Table S1.

One challenge for modeling AAV capsids is that they are huge complexes made of 30,000+ amino acids (aa). In order to reduce computational costs for structure modeling and avoid complications arising from non-specific interactions, we modeled each AAV capsid variant using a single peptide spanning the engineered region (Figure 2A). This peptide (residues 587–594 in the VP1 sequence) includes seven inserted residues and eight contextual residues flanking the insertion. All of these residues are surface exposed and may make direct contact with the receptor in the assembled capsid. Modeling this surface peptide (15 aa) is far less computationally intensive than modeling the entire capsid or even an asymmetric capsid subunit (500+ aa). In addition, compared to the latter, it may improve accuracy by eliminating competing interactions of residues normally buried in inter-subunit interfaces.

To discriminate relatively small differences in receptor-binding propensities of candidate peptides, we modeled the peptides pairwise in competition for the target receptor.^27^^,^^42^ To evaluate the competition results efficiently, we designed a score based on simple atom counting as a rough estimate of the interface free energy between the POI and the receptor in a structure model (Figure 2B). This score, which we term the energetic binding score ($B_{energetic}^{POI}$, simplified as $B_{0}^{POI}$), is a non-negative value calculated from the numbers of contacting and clashing atoms at the interface (Equation 1). Upon analyzing the distribution of $B_{0}^{POI}$ for PHP.eB and AAV9 in our LY6A-binding AAV dataset, we observed an expected disparity in the distribution of the two variants. Specifically, LY6A binder PHP.eB consistently obtained higher $B_{0}^{POI}$ compared to non-binder AAV9 in our competitive modeling results (Figure S2). We describe the detailed rationale behind this score in the section “materials and methods.”

(Equation 1) $$\begin{array}{r} B_{0}^{POI}=B_{energetic}^{POI}=\max\;\left(N_{contact}^{POI}-10^{3}\cdot N_{clash}^{POI},0\right) \end{array}$$

To take full advantage of the information encoded in the competitive models, we further derived a "relative binding score,” inspired by the "specificity strategy" for protein-protein interface design.^43^ The relative score takes the difference between the absolute scores for the POI and competitor peptide (Equation 2), rewarding POIs destabilizing competing peptides’ binding.

(Equation 2) $$\begin{array}{r} \Delta B_{0}^{POI,competitor}=B_{0}^{POI}-B_{0}^{competitor} \end{array}$$

An engineered protein must meet certain geometrical constraints to effectively bind to a membrane receptor (Figure 2C). To utilize this geometrical information, which is likely unused by structure prediction tools, we incorporated two essential constraints for effective binding: the binding angle and the binding depth (Figures 2C–2E).

The first constraint comes from the angle a binding protein can make (Figures 2C and 2D). In modeling a peptide-receptor complex using the extracellular domain of the membrane receptor (e.g., LY6A), most structure predictors (e.g., AF-Multimer) would consider the whole surface of the domain to be accessible by the peptide. However, in biological conditions, the membrane-facing side of the target receptor is inaccessible to the engineered peptide. This polarity of accessibility is a general property of any target receptor that is closely anchored to a larger complex. To account for the potentially huge energy cost of an engineered peptide binding these inaccessible locations, we used a steep polynomial term to penalize peptides that bind to the anchor-facing part of the receptor (Figure 2D, defined in the section “materials and methods” by Equation 6). $B_{0}^{POI}$ is adjusted by this geometrical constraint term, rectified to be non-negative, and $\Delta B_{0}^{POI,competitor}$ is also re-calculated accordingly, yielding new scores $B_{1}^{POI}$ and $\Delta B_{1}^{POI,competitor}$ (Equation 3).

(Equation 3) $$\begin{array}{r} \Delta B_{1}^{POI,competitor}=B_{1}^{POI}-B_{1}^{competitor}=\max\left(B_{energetic}^{POI}+B_{angle}^{POI},0\right)-\max\left(B_{energetic}^{competitor}+B_{angle}^{competitor},0\right) \end{array}$$

The second constraint concerns the binding pocket depth (Figures 2C and 2E). We hypothesized that peptides binding to a deeper pocket on the receptor surface might benefit from longer residence time, which is vital for the efficacy of many therapeutics.^44^ Based on this hypothesis, we included a pocket depth consideration in APPRAISE’s scoring function. We used a relative pocket depth measurement instead of an absolute peptide-receptor distance measurement to avoid possible bias caused by the sizes of different target receptors. We then used an odd polynomial term to reward peptides that insert into deep pockets on the receptor while penalizing peptides that attach to surface humps (Figure 2E, defined in the section “materials and methods” by Equation 7). The addition of the depth term gives us an adjusted score $\Delta B_{2}^{POI,competitor}$ (Equation 4).

(Equation 4) $$\begin{array}{r} \Delta B_{2}^{POI,competitor}=B_{2}^{POI}-B_{2}^{competitor}=\max\left(B_{energetic}^{POI}+B_{angle}^{POI}+B_{depth}^{POI},0\right)-\max(B_{energetic}^{competitor}+B_{angle}^{competitor}+B_{depth}^{competitor},0) \end{array}$$

We compared different versions of scoring methods based on competitive modeling results using AF-Multimer modeling (Figures 2F–2I). Individual matching scores with statistical significance were used to determine wins and losses, and the total matching points in a tournament were used to rank all candidate proteins (section “materials and methods”). We found that simple atom-counting-based $B_{0}^{POI}$ can already differentiate LY6A-binding peptides from non-binders (Figures 2G and 2J). Compared to $B_{0}^{POI}$ alone, the relative score $\Delta B_{0}^{POI,competitor}$ showed improved prediction power, a receiver operating characteristic (ROC) area under the curve (AUC) of 0.800 and an area under precision-recall curve (AUPRC) of 0.756 for the training dataset (Figures 2H–2K). Adding both geometrical terms, $B_{angle}$ and $B_{depth}$, into consideration indeed improved the prediction accuracy of the binding score (Figures 2F–2K), yielding an ROC AUC of 0.838 and an AUPRC of 0.845 (Figures 2J and 2K). Importantly, the improvement in ROC AUC mainly came from the low-false-positive-rate segment of the ROC curve, which is crucial for *in silico* screening of engineered proteins. For clarity, we name the version that considers only the angle constraint (through score $\Delta B_{1}$) APPRAISE 1.1 (Figure S3A) and the version that considers both angle and depth constraints (through score $\Delta B_{2}$) APPRAISE 1.2 (Figure 2I).

We then compared AF-Multimer-based APPRAISE 1.2 with other structure-based peptide affinity ranking methods on the AAV dataset (Figure 2K). With this particular dataset, the model confidence scores pLDDT, pTM, and interface pTM failed to differentiate whether an AAV variant is an LY6A binder, producing worse-than-random prediction (ROC AUC <0.5). This is possibly due to the dynamic nature of the interaction between LY6A-binding AAV variants and the receptor,^40^^,^^41^ which causes the confidence scores of the complex models to be generally low. Meanwhile, APPRAISE 1.2 utilizing ESMFold as the structure prediction engine performed at a comparable level to AF-Multimer-APPRAISE 1.2 (Figure S3B), with an ROC AUC of 0.895 and AUPRC of 0.818 (Figure 2K).

AF-Multimer-APPRAISE 1.2 ranking outperformed all other ranking methods at the low false positive rate end of the ROC curve, with a true-positive rate of 0.714 and no false-positive predictions. The performance with stringent cutoff values is particularly relevant for protein engineering applications, where the goal is typically to identify a few positive binders from many negative, non-binding candidates. The superiority of AF-Multimer-APPRAISE 1.2 in dealing with this kind of imbalanced library is also shown by its highest AUPRC. Because of this, we chose to characterize AF-Multimer-APPRAISE 1.2 further. In the following text, “APPRAISE” will be used to refer to AF-Multimer-APPRAISE 1.2 unless otherwise specified.

### APPRAISE is generally applicable to diverse classes of engineered proteins

To determine the applicability of APPRAISE to different classes of engineered proteins, we applied the method to four classes of engineered protein binders targeting four representative targets for therapeutics.

**Figure 3:**
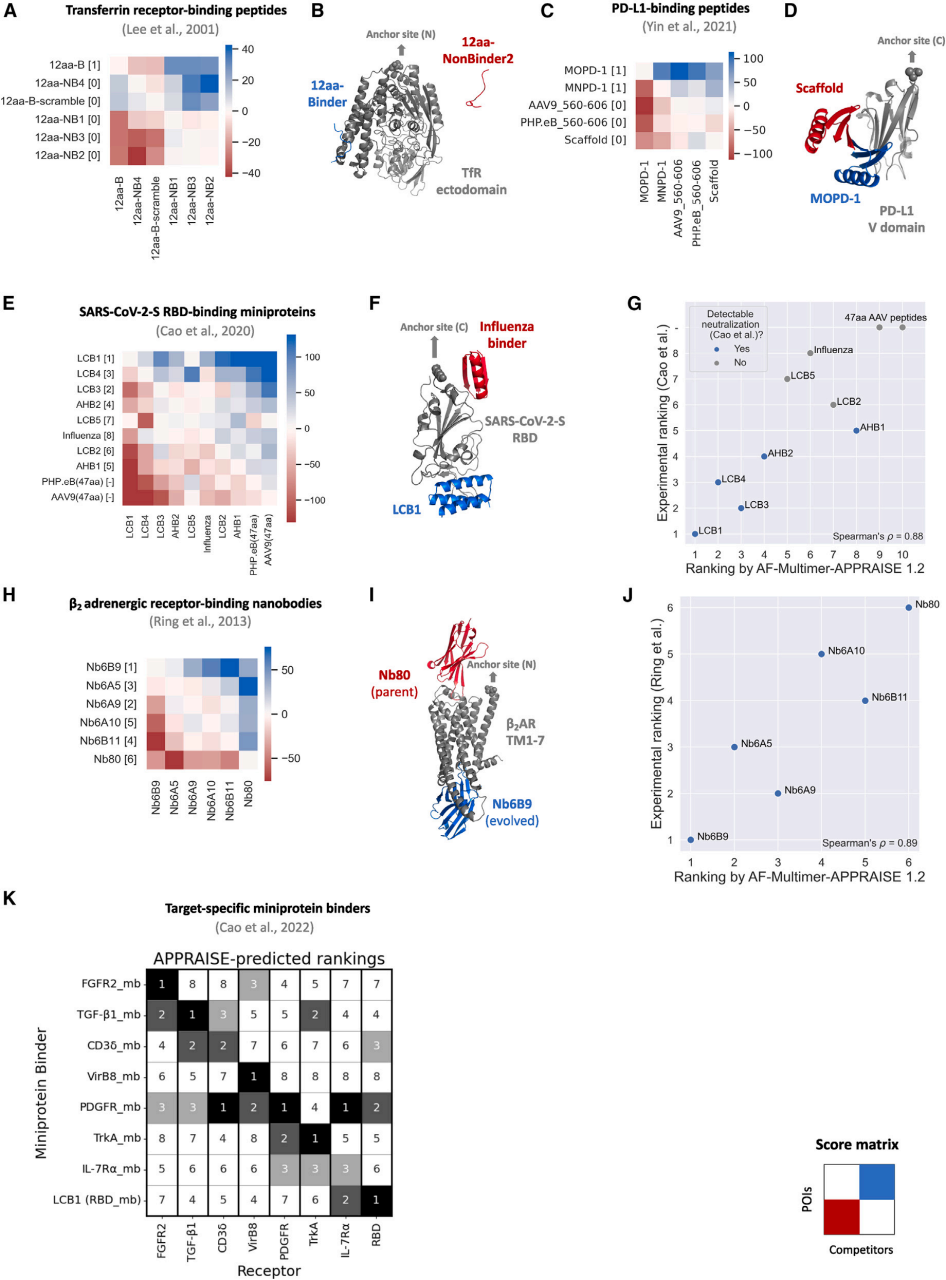
AF-Multimer-APPRAISE 1.2 accurately ranks binding propensities of different classes of engineered proteins (A and B) APPRAISE ranking of transferrin receptor-binding peptides and non-binding control peptides.^4^ (A), Pairwise score matrix and ranking of a panel of 12-aa peptides given by APPRAISE. Bracketed numbers in the row labels are experimentally determined transferrin receptor-binding profiles of each peptide.^4^ (B) A representative AF-Multimer model result of a binding peptide (blue) competing against a non-binding peptide (red) for binding to transferrin receptor. (C and D) APPRAISE ranking of PD-L1-binding peptides and non-binding control peptides.^7^ (C) Pairwise score matrix and ranking of a panel of 47 aa peptides given by APPRAISE. Bracketed numbers in the row labels show the PD-L1-binding profile of each peptide determined either experimentally (for MOPD-1, MNPD-1, and scaffold protein) or by expectation (for AAV9 and PHP.eB).^7^ (D) A representative AF-Multimer model result of MOPD-1 (blue), a designed binding peptide, competing against a non-binding scaffold peptide (red) for binding to PD-L1. (E–G) APPRAISE ranking of SARS-CoV-2-S RBD-binding miniproteins.^5^ (E) Pairwise score matrix and ranking given by APPRAISE. Bracketed rankings in the row labels are determined based on experimentally measured IC_50_ of each miniprotein to neutralize live SARS-CoV-2.^5^ (F) A representative AF-Multimer model result of LCB1 (blue), a SARS-CoV-2-S RBD-binding miniprotein, competing against an influenza virus-binding miniprotein^6^ (red). (G) A scatter plot showing the correlation between APPRAISE-predicted ranking and experimentally measured IC_50_ ranking of all miniproteins tested. Blue points highlight binders that showed the capability of complete neutralization of the SARS-CoV-2 virus in the tested range of concentration *in vitro*. (H–J) APPRAISE ranking of $\beta_{2}$ adrenergic receptor-binding nanobodies.^3^ (H) Pairwise score matrix and ranking given by APPRAISE. Bracketed numbers in the row labels are rankings of experimentally measured binding of each nanobody.^3^ (I) A representative AF-Multimer model result of Nb6B9 (blue), the strongest evolved binder to active $\beta_{2}$ AR, competing against Nb80 (red), the parent nanobody used as the starting point for the evolution. (J) A scatterplot showing the correlation between APPRAISE-predicted ranking and experimentally measured ranking by $\beta_{2}$ AR binding of all nanobodies tested. Each block in the heatmap represents the mean score measured from 10 structural models generated by AlphaFold-Multimer. For comparison, rankings given by AF-Multimer-APPRAISE 1.0, ESMFold-APPRAISE 1.2, and interface pTM of SARS-CoV-2-S RBD-binding miniproteins and $\beta_{2}$ adrenergic receptor-binding nanobodies are shown in Figure S5. (K) A summary of APPRAISE rankings of eight miniproteins^45^ designed to bind to eight different target receptors. Figure S7 displays the score matrices utilized for rankings with individual target receptors. Tables S1 and S2 include sequences and shape parameters of all target receptors. Table S3 includes sequences of all engineered proteins.

We first applied APPRAISE to other short peptide binders (Figures 3A–3D). In the first trial, the method successfully ranked a peptide selected by phage display to bind human transferrin receptor,^4^ a well-characterized BBB receptor, over non-binding counterparts from the same selection^4^ (Figure 3A). In the second trial, we evaluated two 47 aa, rationally designed programmed death-ligand 1 (PD-L1)-binding peptides^7^ against the scaffold and length-matched AAV variable region fragments. Both designed PD-L1-binding peptides were clear winners, with the higher-affinity MOPD-1 peptide topping the list despite a high degree of sequence similarity (Figures 3C, and S4A).

We next tested whether APPRAISE can be used to evaluate larger proteins; for example, computationally designed miniproteins (50–90 aa) that bind to the receptor-binding domain (RBD) of SARS-CoV-2 spike protein^5^ (Figures 3E–3G). Among the designed miniproteins, five can neutralize live SARS-CoV-2 virus *in vitro* with half maximal inhibitory concentration (IC_50_) from 20 pM to 40 nM^5^ The APPRAISE rankings of the five neutralizing miniproteins matched well with their IC_50_ rankings (Spearman’s ρ=0.90, p=0.037; Figure 3G). The predictive accuracy of APPRAISE decreased when non-neutralizing miniproteins^5^ and control AAV fragments were included (Spearman’s ρ=0.88, p<0.001; Figure 3G); nevertheless, the top four binders still remained on the top. In contrast, the ranking given by the interface pTM (ipTM) score of AF-Multimer only achieved a Spearman’s ρ of 0.67 (p=0.035) (Figure S5C).

We also used APPRAISE to rank six nanobodies (120 aa) that were evolved experimentally^3^ with highly similar scaffolds (Figure S4B) to bind to an activated conformation of $\beta_{2}$ adrenergic receptor ($\beta_{2}$ AR), a G-protein-coupled receptor (GPCR) (Figures 3H–3J). APPRAISE correctly found the strongest evolved binder and placed the parent (the weakest binder among all candidates) at the bottom (Figure 3H). The overall predicted ranking correlated well with the ranking from experimentally determined binding readouts^3^ (Spearman’s ρ=0.89, p=0.02; Figure 3J), surpassing the prediction given by the ipTM score of AF-Multimer (Spearman’s ρ=0.49, p=0.329; Figure S5F). Our hypothesis for why APPRAISE is effective in predicting challenging targets is that introducing a competitive protein compels the AlphaFold network to choose a higher-probability binder between two similar options, thereby amplifying the signal. In line with our hypothesis, our evaluation of the binding energy^46^ of AlphaFold predicted models in both the single-POI setting (Figures S6A and S6B) and the competitive-binding setting (Figures S6C and S6D) revealed that the involvement of the competitive protein indeed improved the predictive power of the modeling results. Although competitive modeling has enabled the ranking of nanobodies in this particular instance, it is important to recognize that predicting adaptive immune complexes, particularly larger ones such as immunoglobulin (Ig) G-antigen complexes, still presents a significant challenge. Further advancements in the underlying structure prediction methods will enable APPRAISE to generalize the ranking capability to these challenging targets.

To evaluate the cross-target capabilities of APPRAISE, we used the method to rank eight recently developed miniproteins binding eight different therapeutically significant target receptors.^45^ This ranking included all target receptors with a ligand-binding domain that is smaller than 250 aa in the Cao et al. study. APPRAISE accurately identified the correct binder within the top three in every instance, and, six out of eight times, the correct binder was ranked as the top one (Figures 3K and S7).

We next compared the performance of AF-Multimer-APPRAISE 1.2 to alternative methods on both the miniprotein dataset and the nanobody datasets. AF-Multimer-APPRAISE 1.2 again yielded the most accurate predictions when compared to AF-Multimer-APPRAISE 1.0, ESMFold-APPRAISE 1.2, or interface pTM scores given by AF-Multimer (Figure S5), reflected by higher Spearman’s correlation to experimental rankings. ESMFold-APPRAISE 1.2 failed completely with the miniprotein dataset (Figure S5B). Upon further inspection, we found that the unfolded SARS-CoV-2-S RBD structure in ESMFold-generated complex models can explain the failed ranking prediction.

Without any fine-tuning, AF-Multimer-APPRAISE 1.2 demonstrated consistent prediction ability for ranking all four classes of proteins, including experimentally selected and rationally designed peptides, computationally designed miniproteins, and nanobodies. Realizing the potential general applicability of the APPRAISE method, we have created a web-based notebook interface to make it readily accessible to the protein engineering community (Figure S8, https://tiny.cc/APPRAISE).

### HT-APPRAISE screening can identify novel receptor-dependent capsid variants

**Figure 4:**
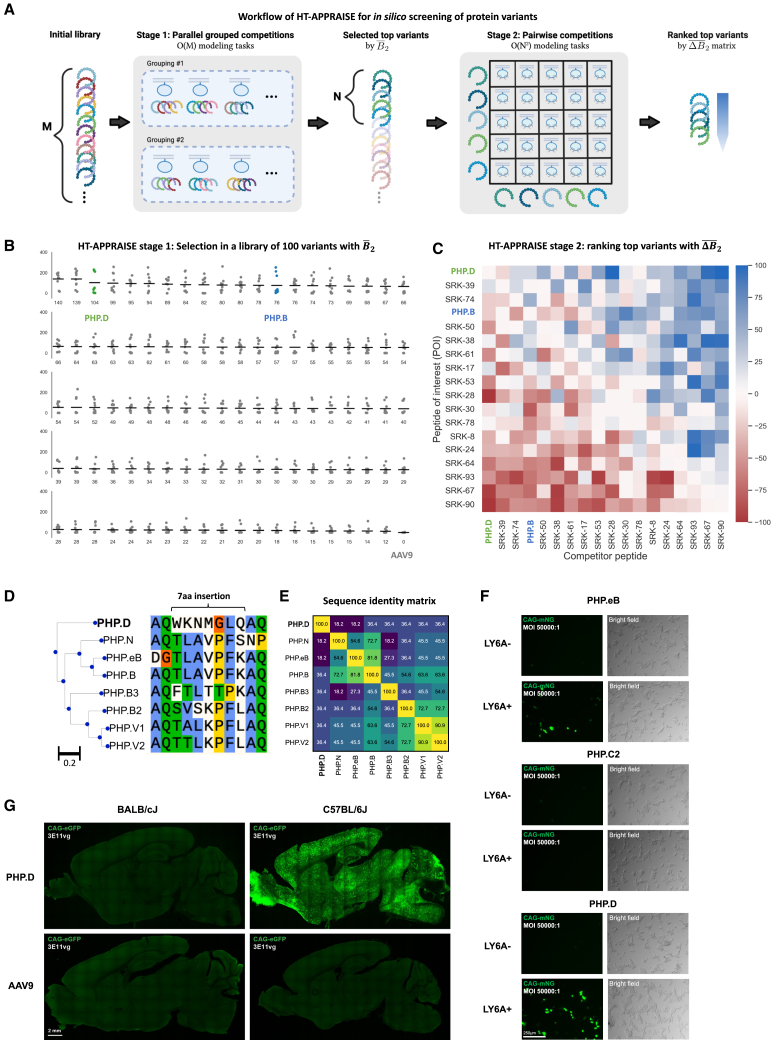
Proof-of-principle HT-APPRAISE screening identifies a LY6A-dependent variant with a distinct sequence (A) A schematic showing the two-stage strategy for *in silico* screening of a variant library. In the first stage, M variants of interest are randomly pooled into groups of four and compete for target receptor binding. At least two parallel groupings are used to reduce bias. Each peptide’s mean absolute binding score in the pool competitions is used for selecting the top N variants. In the second stage, the top N variants compete pairwise using standard APPRAISE for a more accurate ranking. (B and C) Results from a proof-of-principle screening with 100 AAV9-based variants, including the wild-type control and variants with 7-aa insertions. Using a standard random algorithm, a total of 97 variants were picked from a list of 9,000 variants^2^ that demonstrated higher brain enrichment than the wild-type AAV9 after one round of screening in C57BL/6J mice (Round 2 library in Ravindra Kumar et al.^2^). PHP.B and PHP.D, two known brain-transducing capsids, and wild-type AAV9, were spiked into the library. Table S4 shows the peptide sequences used in the screening. (B) Stage 1 ranking result. Dots indicate absolute binding scores measured from individual structure models. Horizontal bars indicate the mean scores of each variant. Scores of PHP.D (ranked third), PHP.B (ranked $13^{th}$), and AAV9 (ranked $100^{th}$) are highlighted. (C) Stage 2 ranking result. Rows corresponding to scores of PHP.D (ranked first) and PHP.B (ranked fourth) are highlighted. Each block in the heatmap represents the mean score measured from 10 structural models generated by AlphaFold-Multimer. (D–G) Characterization of PHP.D, a variant that tops the *in silico* screening. (D) Sequence alignment and phylogenetic tree of known LY6A-dependent brain-transducing AAV variants. The sequence of PHP.D is very different from all other variants. The alignment and sequence distances were generated with Clustal Omega.^47^ The colored alignment is plotted with Snapgene software. Blue, conserved hydrophobic residues; green, conserved hydrophilic residues; orange or yellow, conserved unique residues (glycine or proline). (E) Sequence identity matrix of the LY6A-dependent variants. (F) *In vitro* infectivity assay in HEK293T cells. PHP.D and PHP.eB showed LY6A-enhanced transduction, while the negative-control PHP.C2 did not show LY6A-enhanced transduction. AAV capsids carrying a fluorescent protein expression cassette were applied to HEK293T cells either transfected with LY6A or not at 5 × 10^8^ viral genomes (vg) per well in a 96-well plate. Images were taken 24 h after transduction (*n* = 3 per condition). Scale bar, 250 μm. (G) *In vivo* brain transduction by PHP.D vs. AAV9 in two different mice strains. PHP.D shows efficient brain transduction only in C57BL/6J, the strain with brain LY6A expression, but not in BALB/cJ, the strain without brain LY6A expression. AAVs carrying CAG-mNeonGreen transgene were injected intravenously at 3 × 10^11^ vg per animal, and the tissues were harvested and imaged 3 weeks after injection (*n* = 3 per condition). Scale bar, 2 mm.

We next adapted APPRAISE for *in silico* screening. The computational cost in the pairwise competition mode grows quadratically with the number of input variants, which is unsuitable for high-throughput screening. To address this scalability issue, we designed a two-stage screening strategy named high-throughput (HT)-APPRAISE (Figure 4A). The first stage aims to shrink the size of the variant library using a less accurate yet more scalable strategy. Variants are randomly pooled into groups and compete for target receptor binding. The variants are then ranked by their absolute score ${\bar{B}}_{2}^{POI}$. The number of pooled competitions grows linearly with the number of variants in the starting library, making the first stage of HT-APPRAISE suitable for larger libraries. In the second stage, the top variants selected from the first stage compete pairwise, yielding a matrix of ${\bar{\Delta B}}_{2}^{POI,competitor}$ and a more accurate ranking.

We used our HT-APPRAISE *in silico* screening to find LY6A binders in a library of 100 capsid variants (Figures 4B and 4C). This library is composed of 97 capsid variants randomly chosen from a list of 9,000 variants showing superior brain enrichment in C57BL/6J mice compared to the wild-type AAV9 capsid^2^ as well as three spiked-in capsids: the variants PHP.B and PHP.D, and the wild-type AAV9 (Table S4). PHP.D is a brain-transducing capsid identified in a recent directed evolution campaign in our lab, the relevant BBB receptor for which was unknown.

The HT-APPRAISE screening took 24 h using three parallel Google Colaboratory GPU sessions and a laptop computer. In both stages of the HT-APPRAISE, the most time-consuming step was the structural prediction, which took approximately 0.1–1 GPU minute per peptide-LY6A model (a complex made of 114 aa total). In comparison, the time cost for structural analysis was negligible, taking less than 1 s per model on a CPU.

After the first stage of screening, both PHP.D and PHP.B appeared in the top 15% of the library (ranked by ${\bar{B}}_{2}^{POI}$) (Figure 4B). In the second stage, the top 18 capsids were ranked using pairwise APPRAISE (Figure 4C). PHP.D and PHP.B were the first and the fourth in the final ranking, respectively.

The most intriguing aspect of PHP.D’s result is that its variable region bears little sequence similarity to any of the LY6A-dependent variants used to develop the APPRAISE method (Figures 4D and 4E). To confirm this prediction result, we experimentally tested PHP.D’s LY6A dependency. An *in vitro* viral infection assay showed that PHP.D indeed exhibits LY6A-enhanced transduction of HEK293T cells (Figure 4F). In addition, *in vivo* systemic delivery of PHP.D packaging a ubiquitously expressed fluorescent protein revealed that the brain transduction capability of this capsid variant is restricted to LY6A-expressing mouse strains (Figure 4G). The ability of HT-APPRAISE to identify binders with distinct sequences highlights the generalizability of the physics-informed, sequence-structure/structure-function strategy.

## Discussion

Here, we describe APPRAISE, a structure-based, physics-informed method that accurately ranks target-binding propensities of engineered proteins. APPRAISE uses a competitive, pairwise modeling strategy to capture affinity differences between even proteins with similar sequences and takes into account biophysical and geometrical principles.

The competition-based structure modeling strategy aims to address the challenge of assessing small differences in binding affinity with high accuracy. This challenge was highlighted by a recent benchmarking study using AF2 models for molecular docking of small-molecule antibiotic candidates. The authors reported that the prediction power of the direct physics-based scoring is no better than a random model.^48^ By contrast, a competition-based modeling strategy might help cancel the shared noise and amplify the signal arising from the small affinity differences in certain cases, such as the nanobody dataset we tested (Figure S6). The competition setup, in many cases, forces the structure prediction neural network to put only the more probable binder close to the target receptor (Figures 3D and 3I), converting a small probabilistic difference into a binary output.

The generalizability of AF-Multimer-APPRAISE is shown by its accurate ranking of five different classes of engineered proteins and 12 different target receptors. This generalizability may be grounded in the physical principles learned by AlphaFold.^27^^,^^49^ For example, consistent with the report of Chang and Perez,^27^ we also observed a recurring trend in our tests with AF-Multimer where binders ranked at the top are frequently predicted with secondary structures, which may indicate a stable binding interface. While we were revising this manuscript, APPRAISE was applied in various scenarios, extending beyond the examples presented in this manuscript. For instance, in a recent study uncovering previously unknown BBB receptors used by engineered AAVs, APPRAISE helped classify the AAVs based on their receptor specificity.^50^

A key feature of APPRAISE is that its analysis module uses only information stored in the 3D coordinates, making the modular pipeline compatible with other computational tools for protein engineering. For example, the AlphaFold-Multimer used in APPRAISE can be replaced by any current or future structure prediction tool. Moreover, APPRAISE can be an orthogonal validation tool for structure-based protein design methods,^51^^,^^52^^,^^53^ particularly those that rely on optimization of predicted confidence scores.^54^

The scalable, two-stage HT-APPRAISE strategy we designed provides a path for *in silico* screening of protein candidates for target receptor binding (Figure 4). Such screening can help prioritize leading candidates during drug discovery, reducing the time, financial, and environmental costs of experimental validation. For example, the computational tasks needed to screen 100 AAV variants that we presented here (Figure 4) could be completed within 24 h with research-grade computational resources. *In vivo* characterization of the capsids at a comparable scale would have taken several months.

As a competition-based ranking method, APPRAISE faces several intrinsic limitations. One such limitation is that APPRAISE only outputs the relative, not the absolute, probability of binding. Therefore, unless there are positive controls with known binding to compare against, a variant’s position at the top of the ranking does not indicate that the variant has an experimentally detectable binding affinity. Another limitation lies in APPRAISE’s assumption that the binding of competing proteins is mutually exclusive. Counterexamples arise if the competing proteins exhibit cooperative binding or attach to epitopes situated at a considerable distance. Furthermore, certain candidate proteins may exhibit a tendency to interact with one another rather than with the designated target receptor. Additionally, the geometrical scores utilized in APPRAISE 1.1+ were computed assuming the target receptor has a predominantly convex structure. Thus, these scores are most effective when applied to single protein domains with convex shapes.

Other limitations of APPRAISE may arise from the protein structure prediction engine that it relies on. For example, ESMFold-APPRAISE fails when the language-model-based structure prediction tool cannot properly fold the protein in a complex (Figure S5B). At the same time, AF-Multimer-APPRAISE results can be biased by the specific selection of multiple sequence alignments due to the dependence on co-evolutionary information by AF-Multimer. So far, the APPRAISE pipeline’s ability to accurately rank IgG antibodies is limited due to the difficulty of predicting antibody-antigen complex structures. Moreover, the accuracy and speed of APPRAISE may be compromised when the modeled proteins contain long disordered regions or large domains that are unnecessary for binding. As a result, pre-screening of several truncated protein constructs for minimal folding domains with the particular structure prediction tool (analogous to the common practice in structural biology) is always helpful. Additionally, the APPRAISE method is ineffective in ranking weak binders in a pool (e.g., Figure 3G), perhaps because the predicted structures do not offer many opportunities for meaningful interaction, resulting in near-zero competition scores. Fortunately, this should not be a practical concern for most protein engineering applications since the most valuable candidates usually bind with higher affinities. Considering these limitations, it is essential to conduct spot checks on model results to confirm their physical soundness.

While APPRAISE has succeeded in ranking the binding propensities of different protein variants, its accuracy and speed could be further improved. For example, the parameters of APPRAISE 1.2’s scoring function have only been minimally tuned to physically justified orders of magnitude to decrease the risk of over-fitting (section “materials and methods”). With further fine-tuning of parameters and the ever-growing power of protein structure prediction, the APPRAISE method promises to streamline the process of engineering protein-based therapeutics.

## Materials and methods

### Structure modeling

#### Modeling of peptide-receptor complexes using AF-Multimer

Peptide-receptor models are modeled using ColabFold (Python package index: AlphaFold-ColabFold 2.1.14), an implementation of integrated multiple sequence alignment generation with MMseqs2 and structure modeling with AF-Multimer-v2.^14^^,^^15^^,^^55^^,^^56^^,^^57^^,^^58^ First, batches of ∗.fasta files containing combined target receptor sequences (Tables S1 and S2) and peptide sequences for the pairwise competition or pooled competition, where the ":" symbol separates the protein chains, are prepared using the function (appraise.input_fasta_prep.get_complex_fasta()). Second, the ∗.fasta files are used as input files for the "batch" Jupyter notebook in the ColabFold package, and the notebook is run on Google Colaboratory using a V100 SXM2 16GB GPU or an A100 SXM4 40GB GPU. The settings used for the modeling are listed below. In this study, all complex structures were modeled with templates.

msa_mode = "MMseqs2 (UniRef+Environmental)"

num_models = 5

num_recycles = 3

stop_at_score = 100

use_custom_msa = False

use_amber = False

use_templates = True

model_type = "auto" #or "alphafold2_multimer_v2"

#### Modeling of peptide-receptor complexes using ESMFold

To model the peptide-receptor complexes using ESMFold, a process analogous to the one employed for AF-Multimer modeling is implemented. First, batches of ∗.fasta files containing combined target receptor sequences (Tables S1 and S2) and peptide sequences for the pairwise competition or pooled competition, where the protein chains are separated by a poly-glycine linker (30 glycine residues), are prepared using the same Python function (get_complex_fasta()) mentioned above. Second, the ∗.fasta files are used as input files for a custom Jupyter notebook with codes adapted from the ColabFold package for batch modeling using ESMFold, and the notebook is run on Google Colaboratory using an A100 SXM4 40GB GPU. The custom Colab notebook is included in the APPRAISE package:

appraise/misc_utilities/ColabFold_ESMFold_batch_run.ipynb.

### Physics-informed analysis of individual structure models

The output folder containing ∗.pdb files generated by alphafold-colabfold is downloaded to a local computer for processing. Key parameters in a predicted structure model are measured, and the measurements are used to generate binding scores for each peptide in a model.

#### Automated quantification of the peptide-receptor models

The structure models are analyzed using PyMOL 2.3.3 using a custom PyMOL script. Briefly, the script loads all ∗.pdb models in a directory; extracts metadata from the file names; and measures the relevant contact atom numbers, angles, and distances. The measurements are saved as an ∗.csv file. The custom PyMOL script is included in the APPRAISE package:

appraise/pymol_quantify_peptide_binding.py.

#### Measurement of the $R_{minor}$ of the receptor hull

The shape parameter $R_{minor}$ of a target receptor, which is necessary for APPRAISE 1.2, is obtained by measuring the shape parameters of an AlphaFold-modeled receptor structure. Briefly, the monomeric receptor (Table S1) is modeled using ColabFold (Python package index: alphafold-colabfold 2.1.14). The top model is then analyzed using HullRad v8.1^59^ to obtain its major axis diameter $D_{\max}$ and aspect ratio P. $R_{minor}$ is then calculated using the formula $R_{minor}=D_{\max}/P/2$. Before the analysis, $R_{minor}$ measurement is manually added as a column to the pandas dataframe storing PyMOL measurements with the column "R_minor.”

#### Construction and calculation of $B_{energetic}$

We defined a contact atom as a non-hydrogen atom of either the target receptor or the peptide within 5 Å of the binding partner in the peptide-receptor model since atoms within this distance cutoff are responsible for most protein-protein interactions.^60^ We defined a clashing term as the number of non-hydrogen atoms in the peptide that are within 1 Å of the receptor since this distance is smaller than the typical diameter of an atom and can cause a huge van der Waals strain. To find the suitable weight for the clashing term, we estimated the relative energy scales using Lennard-Jones’ potential. We concluded that an order of magnitude of $10^{3}$ should be justified (Equation 1). Since most interfaces between the engineered peptide and the receptor have up to a few hundred non-hydrogen atoms (tens of residues) in the interface, this heavy weight for the clashing atom practically sets the $B_{0}^{POI}$ of any peptide with steric clashing against the receptor to 0. Thus, Equation 1 is practically equivalent to

(Equation 5) $$\begin{array}{r} B_{energetic}^{peptide}=\left\{ \begin{array}{ll} N_{contact}^{peptide}, & \text{if}\;N_{clash}^{peptide}=0\\ 0, & \text{if}\;N_{clash}^{peptide}>=1 \end{array}\right. \end{array}$$

#### Construction and calculation of geometrical scores

The binding angle θ is defined as the angle between the vector from receptor center of mass to receptor anchor $\vec{OA}$ and the vector from receptor center of mass to peptide center of mass $\vec{OC^{\prime }}$ (Figure 2C. Note that the peptide center of mass $C^{\prime }$ is usually very close to the deepest point C, and therefore point C and point $C^{\prime }$ are undifferentiated in this schematic). A steep function is used to penalize inaccessible binding angles that are close to the anchor point:

(Equation 6) $$\begin{array}{r} B_{angle}^{peptide}=\left\{ \begin{array}{ll} -10^{3}\cdot {\left(1-\frac{\theta }{\frac{\pi }{2}}\right)}^{10}, & \text{if}\;\theta <\frac{\pi }{2}\\ 0, & \text{if}\;\frac{\pi }{2}<=\theta <=\pi \end{array}\right. \end{array}$$

The definition of binding depth d is a simplification of previously defined travel depth^61^: we first calculate the hydrodynamic radius of the hull of the receptor at the minor axis ($R_{minor}$) using HullRad,^59^ and then take the difference of the distance between the closest point on the peptide to the receptor center and $R_{minor}$. The ratio between the difference and $R_{minor}$ is defined as the depth. In other words, binding depth $d=\frac{∥OB∥-∥OC∥}{∥OB∥}$ where ∥OB∥ is the minor axis radius (in Å) of the receptor hull when considering it as an ellipsoid (Figure 2C), and ∥OC∥ is the distance (in Å) between the center of mass of the receptor and the closest point on the peptide (Figure 2C). An odd polynomial function is used to construct the score to reflect both the positive effect of a deep binding pocket and the negative effect of a convex binding site:

(Equation 7) $$\begin{array}{r} B_{depth}^{peptide}=10^{2}\cdot d^{3} \end{array}$$

#### Calculation of scores for each peptide in a model

The total binding scores for each peptide in a model are calculated using Equations 1, 2, 3, and 4 in the main text.

#### Generation of the score matrix and a ranking

The total binding scores of a POI vs. a competitor across replicate models are averaged to get ${\bar{\Delta B}}^{POI,competitor}$ (${\bar{\Delta B}}_{0}^{POI,competitor}$ for APPRAISE 1.0, ${\bar{\Delta B}}_{1}^{POI,competitor}$ for APPRAISE 1.1, or ${\bar{\Delta B}}_{2}^{POI,competitor}$ for APPRAISE 1.2). In this study, we conducted each competition using two different inputs with reversed orders (POI 1-POI 2 and POI 2-POI 1). Consequently, when AlphaFold-Multimer was used, 10 independent models were generated for each competition because AlphaFold-Multimer gives five different predictions for each input, while ESMFold produced two different models for each competition. These averaged competition scores are then used to create a matrix and are plotted as a heatmap.

In the final score matrix, the POIs are ranked using a point-based round-robin tournament system^62^ to avoid the bias caused by individual competitions with unusually high scores. Briefly, each ${\bar{\Delta B}}^{POI,competitor}$ in the matrix is considered as the match result between a POI and a competitor. A POI gains one point for winning over each match and loses one point for losing each match. In the cases when $\left|{\bar{\Delta B}}^{POI,competitor}\right|$ does not reach the threshold of p<0.05 using a one-sample, two-sided, Student’s t test (degree of freedom = 9), the match is called a tie, and the POI gets 0 points from the match.

### Experimental validations

#### *In vitro* infectivity assay

HEK293T (ATCC, CRL-3216) cells were seeded in six-well plates at 80% confluency and maintained in Dulbecco’s modified Eagle’s medium (DMEM) supplemented with 5% fetal bovine serum (FBS), 1% non-essential amino acids (NEAAs), and 100 U/mL penicillin-streptomycin at 37°C in 5% CO_2_. Cells were transiently transfected with 2.53 μg of plasmid DNA encoding an expression cassette for the LY6A receptor. The following day, receptor-expressing cells were transferred to black, clear-bottom, 96-well plates at 20% confluency and maintained in FluoroBrite DMEM supplemented with 0.5% FBS, 1% NEAA, 100 U/mL penicillin-streptomycin, 1× GlutaMAX, and 15 mM HEPES at 37°C in 5% CO_2_. Engineered AAV variants packaging a CAG-mNeonGreen transgene were dosed in triplicate at 5E8 viral genomes (vg) per well once the cells were attached. Plates were imaged 24 h after AAV was introduced to cells with a Keyence BZ-X700 using a 4× objective and NucBlue Live ReadyProbes Reagent (Hoechst 33342) to autofocus each well.

### *In vivo* mouse experiment

For all the experiments performed in this study, the animals were randomly assigned, and the experimenters were not blinded while performing the experiments unless mentioned otherwise. All animal procedures in mice were approved by the California Institute of Technology Institutional Animal Care and Use Committee (IACUC), Caltech Office of Laboratory Animal Resources (OLAR), and were carried out in accordance with guidelines and regulations.

For the profiling of the novel AAVs in C57BL/6J mice (The Jackson Laboratory, 000664) and BALB/cJ mice (The Jackson Laboratory, 000651), the AAV vectors were injected intravenously via the retro-orbital route to 6- to 8-week-old adult mice at a dose of 3 × 10^11^ vg per mouse. Retro-orbital injections were performed as described previously.^63^ To harvest the tissues of interest after 3 weeks of expression, the mice were anesthetized with Euthasol (pentobarbital sodium and phenytoin sodium solution, Virbac AH) and transcardially perfused using 50 mL of 0.1 M phosphate-buffered saline (PBS) (pH 7.4) followed by 50 mL of 4% paraformaldehyde (PFA) in 0.1 M PBS. The organs were collected and post-fixed 24 h in 4% PFA at 4°C. Following this, the tissues were washed with 0.1 M PBS and stored in fresh PBS-azide (0.1 M PBS containing 0.05% sodium azide) at 4°C. Before imaging, the 100-μm tissue slices were cut using a Leica VT1000S. Brain images were acquired with a Zeiss LSM 880 confocal microscope using a Plan-Apochromat 10 × 0.45 M27 (working distance, 2.0 mm) objective. Zen Black 2.3 SP1 was used to process the images.

## Data and code availability

The authors confirm that the data supporting the findings of this study are available within the article and its supplemental materials.

The plasmid expressing the AAV-PHP.D capsid reported in this manuscript is deposited in Addgene (Addgene: 197055).

All code is open-source through an Apache-2.0 license and available in a GitHub repository:

github.com/GradinaruLab/APPRAISE

In addition, APPRAISE is made accessible through a web-based notebook interface using Google Colaboratory. The notebook can be found in the GitHub repository above or be directly accessed through the following short link:

tiny.cc/APPRAISE

The Colab-APPRAISE notebook includes pre-filled templates that can be used to demonstrate the workflow of APPRAISE. More demos can be found under the demo folder in the GitHub repository.

## Supplemental information

Document S1. Figures S1–S8 and Tables S1–S4

Document S2. Article plus supplemental information
