# Supplementary material for: Fast, accurate ranking of engineered proteins by target-binding propensity using structure modeling
Source: Mol Ther. 2024 Apr 6;32(6):1687–700. doi: 10.1016/j.ymthe.2024.04.003 (PMC11184338; doi:10.1016/j.ymthe.2024.04.003)
Supplement: Document S1. Figures S1–S8 and Tables S1–S4 [file mmc1.pdf]

YMTHE, Volume *32*

Supplemental Information

Fast, accurate ranking of engineered proteins by target-binding propensity using structure modeling

Xiaozhe Ding, Xinhong Chen, Erin E. Sullivan, Timothy F. Shay, and Viviana Gradinaru

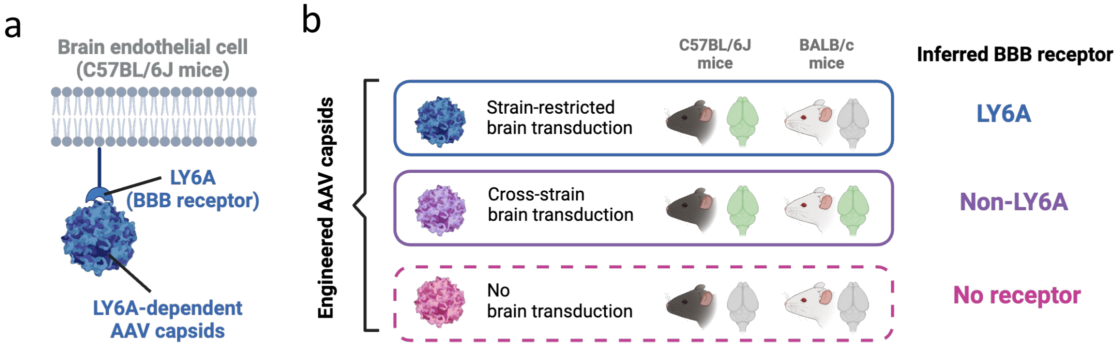

c

Variant name	Ref.	Modeled peptide sequence	C57BL/6J brain?	BALB/c brain?	Inferred LY6A dependency
PHP.B	Deverman et al.	AQTLAVPFKAQAQTG	✓	×	True*
PHP.eB	Chan et al.	DGTLAVPFKAQAQTG	✓	×	True**
PHP.B2	Deverman et al.	AQSVSKPFLAQAQTG	✓	×	True*
PHP.B3	Deverman et al.	AQFTLTTPKAQAQTG	✓	×	True*
PHP.N	Kumar et al.	AQTLAVPFSNPAQTG	✓	×	True
PHP.V1	Kumar et al.	AQTALKPFLAQAQTG	✓	×	True
PHP.V2	Kumar et al.	AQTTLKPFLAQAQTG	✓	×	True
PHP.C1	Kumar et al.	AQRYQGDSVAQAQTG	✓	✓	False
PHP.C2	Kumar et al.	AQWSTNAGYAQAQTG	✓	✓	False
PHP.C3	Kumar et al.	AQERVGFAQAQAQTG	✓	✓	False
AAV9	Gao et al.	AQ-------AQAQTG	×	×	False**
MacPNS1	Chen et al.	AQPHEGSSRAQAQTG	×	-	False
MacPNS2	Chen et al.	AQPNASVNSAQAQTG	×	-	False
NB-1	Kumar et al.	AQSLDTTKGAQAQTG	×	-	False
NB-2	Kumar et al.	AQESRGLSLAQAQTG	×	-	False
NB-3	Kumar et al.	AQMIRGLDTAQAQTG	×	-	False
NB-4	Kumar et al.	AQNGDLTRSAQAQTG	×	-	False
NB-5	Kumar et al.	AQTKREIDYAQAQTG	×	-	False
NB-6	Kumar et al.	AQMHHGMEIAQAQTG	×	-	False
NB-7	Kumar et al.	AQTPRMDTFAQAQTG	×	-	False
NB-8	Kumar et al.	AQVHRDLNFAQAQTG	×	-	False
NB-9	Kumar et al.	AQTARKEEFAQAQTG	×	-	False

* LY6A binding had been confirmed by cell binding assay

** LY6A dependency had been biochemical and genetic assays

Figure S1 Prior experimental studies revealing the receptor dependency of some brain-transducing AAV variants. **a)** A schematic showing an AAV capsid binding to a blood-brain barrier (BBB) receptor that is only expressed at a high level on the endothelial cells of certain mouse strains[37–39]. **b)** A schematic showing how we can infer whether an AAV capsid can use LY6A, a mouse BBB receptor, by characterizing its brain transduction across different strains. A capsid with strain-restricted brain transduction in C57BL/6J mice is likely LY6A-dependent, while a capsid with cross-strain brain transduction or which does not transduce the brain is not LY6A-dependent. **c)** A table summarizing all 22 capsids used in Figure 2 with their source literature, sequence, brain transduction profile, and inferred LY6A dependency.

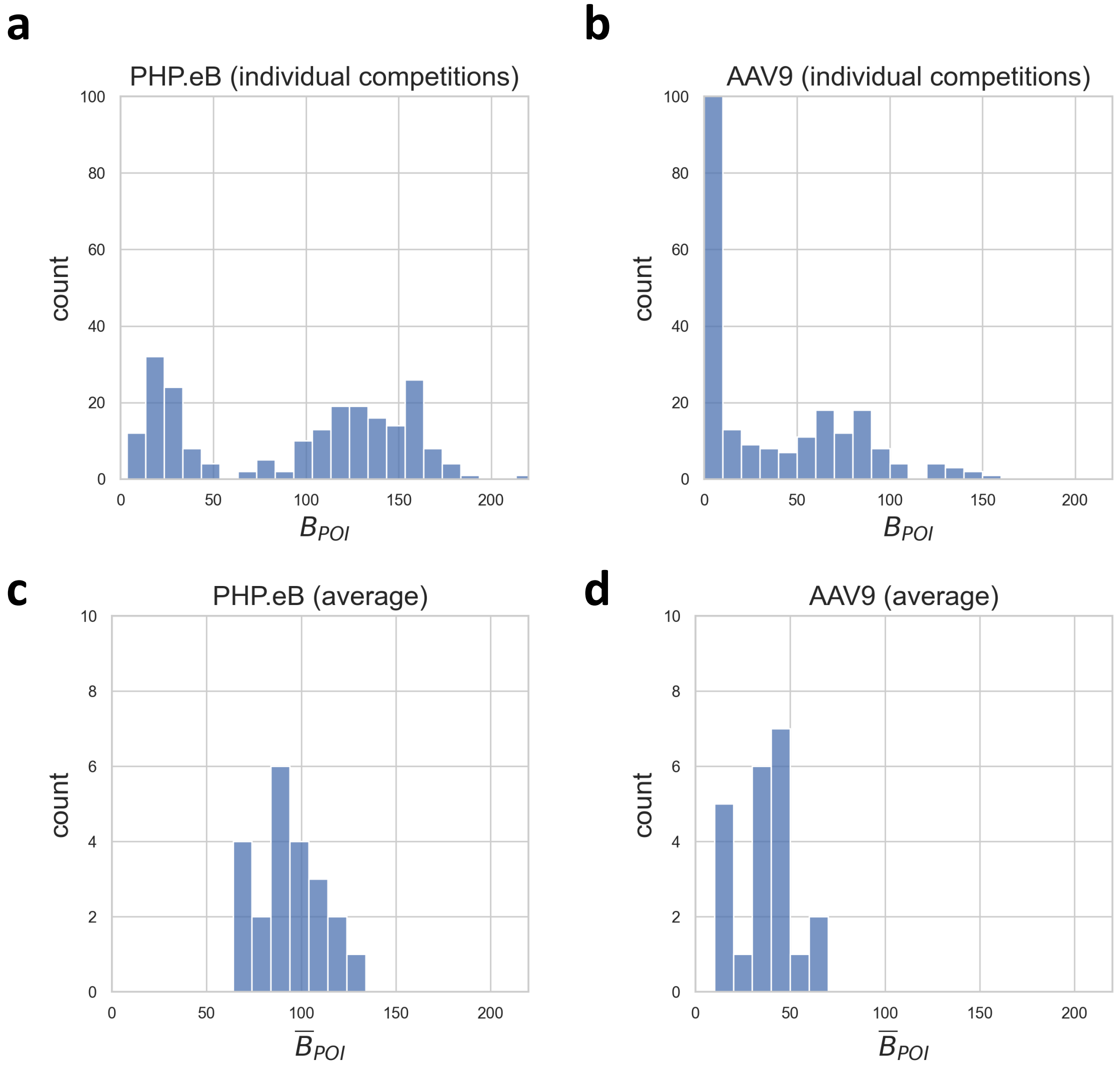

Figure S2 Distributions of interface energy scores in example proteins of interest. Histograms showing distributions of **a, b)** B_0^{POI} and **c, d)** $\overline{B}_0^{POI}$ averaged across replicates for a LY6A binder, PHP.eB, and a non-binder, AAV9, from the AAV dataset.

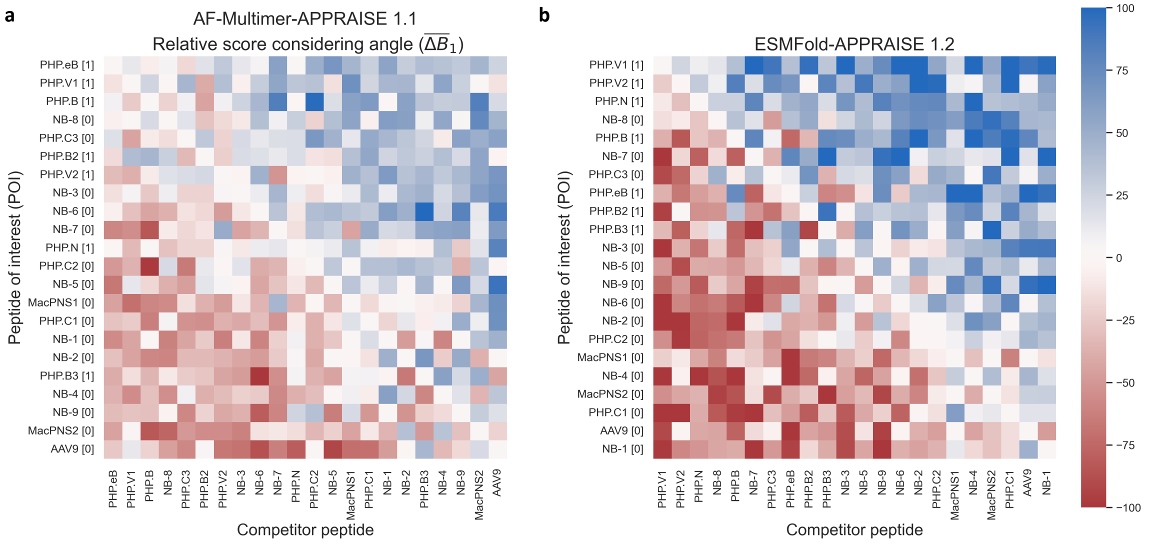

Figure S3 Heatmaps representing score matrices of AF-Multimer-APPRAISE 1.1 and ESMFold-APPRAISE 1.2.

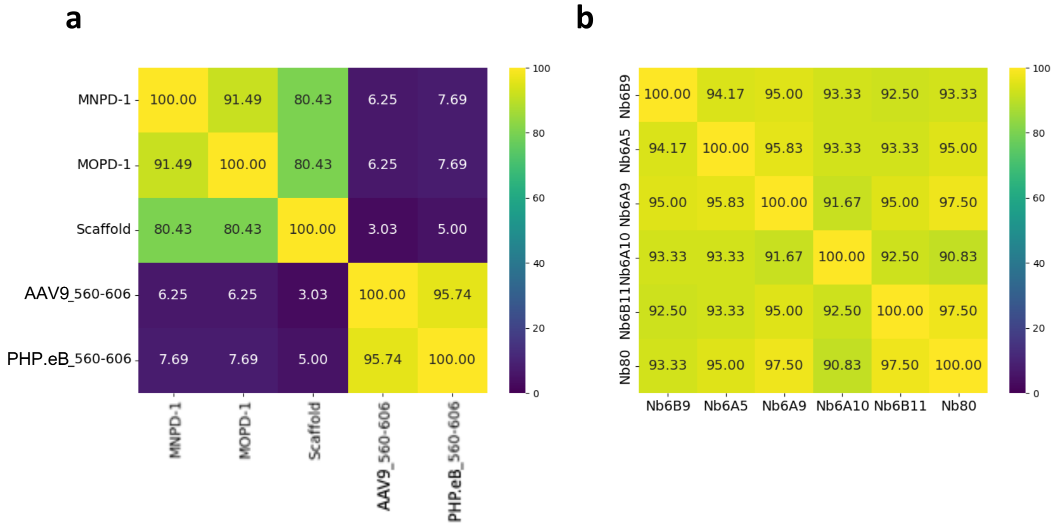

Figure S4 Sequence identity between some engineered proteins. Sequence identity matrix generated with Clustal Omega[46] for **a)** PD-L1-binding peptides and **b)** β_2 adrenergic receptor-binding nanobodies, demonstrating that these proteins share similar sequences.

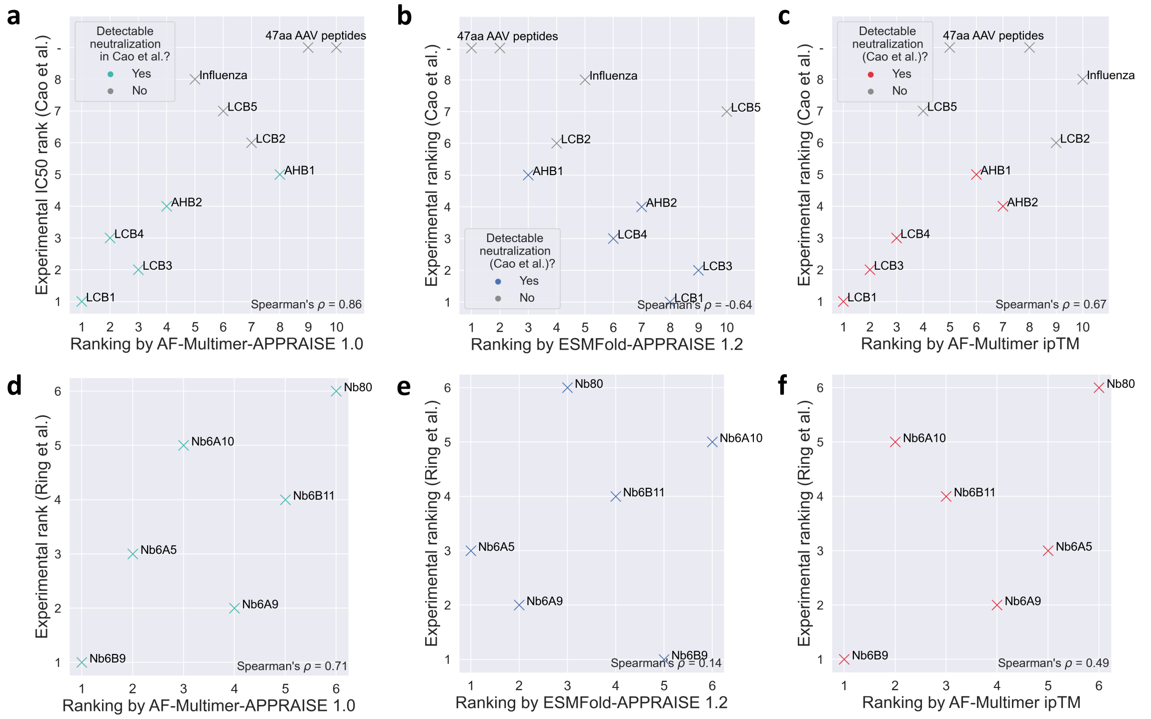

Figure S5 Ranking protein binders using alternative methods. Rankings of two groups of peptides analyzed in Figure 3g, j based on **a, d)** AF-Multimer-APPRAISE 1.0, **b, e)** ESMFold-APPRAISE 1.2, and **c, f)** interface pTM given by AlphaFold-Multimer.

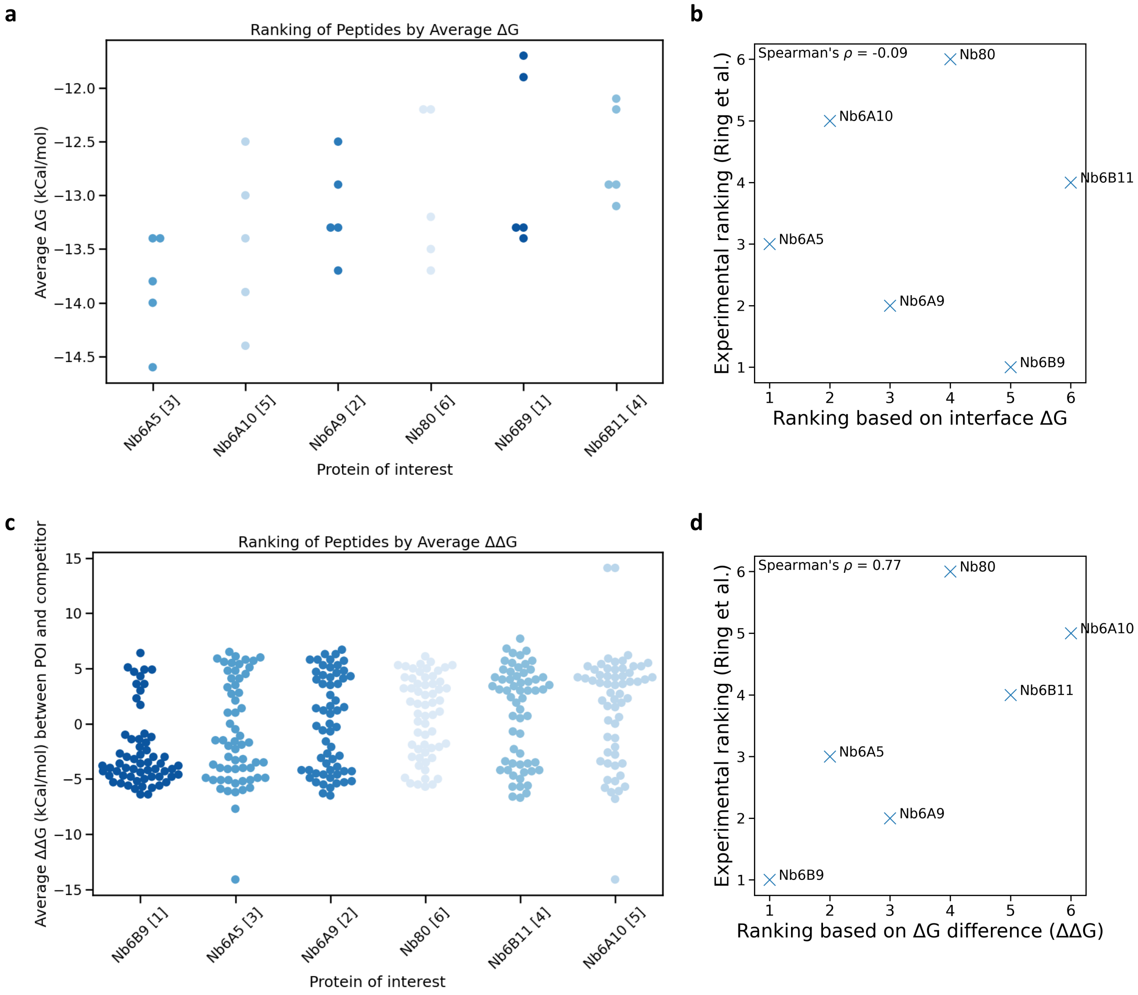

Figure S6 Binding energy analysis of AlphaFold predicted nanobody complex models. a) PRODIGY-predicted interface ΔG[47] of individual nanobodies in complex with the target β_2AR. **b)** Correlation between the ranking given by interface energy and the experimental affinity ranking[3]. **c)** Difference in PRODIGY-predicted interface ΔG[47] of nanobodies modeled competitively in complex with the target β_2AR. **d)** Correlation between the ranking given by difference in interface energy and the experimental affinity ranking[3].

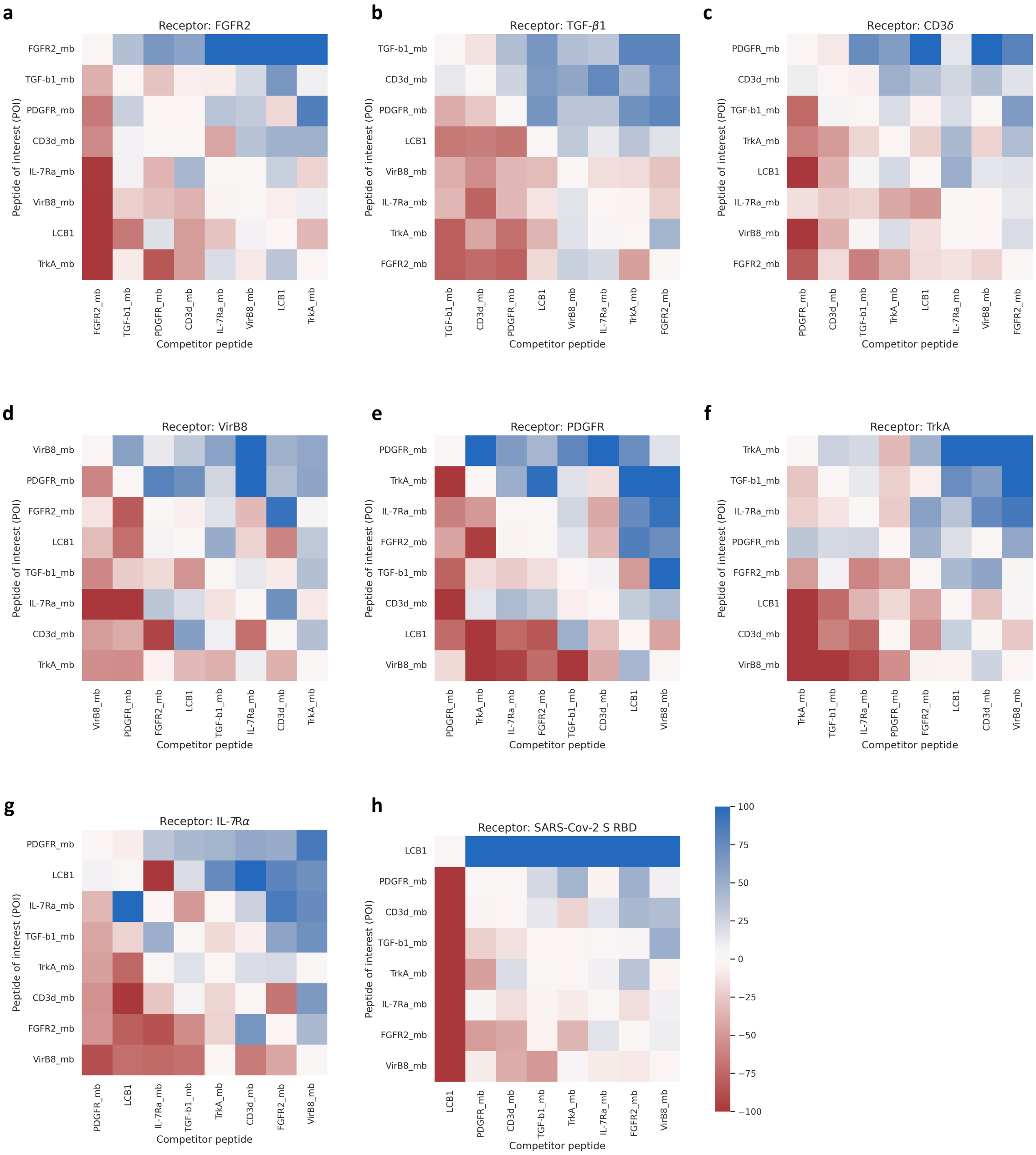

Figure S7 Score matrices for APPRAISE rankings of miniprotein binders with individual target receptors. Detailed rankings of miniproteins with individual receptors summarized in Figure 3k using AF-Multimer-APPRAISE 1.2. The miniprotein sequences were from[45].

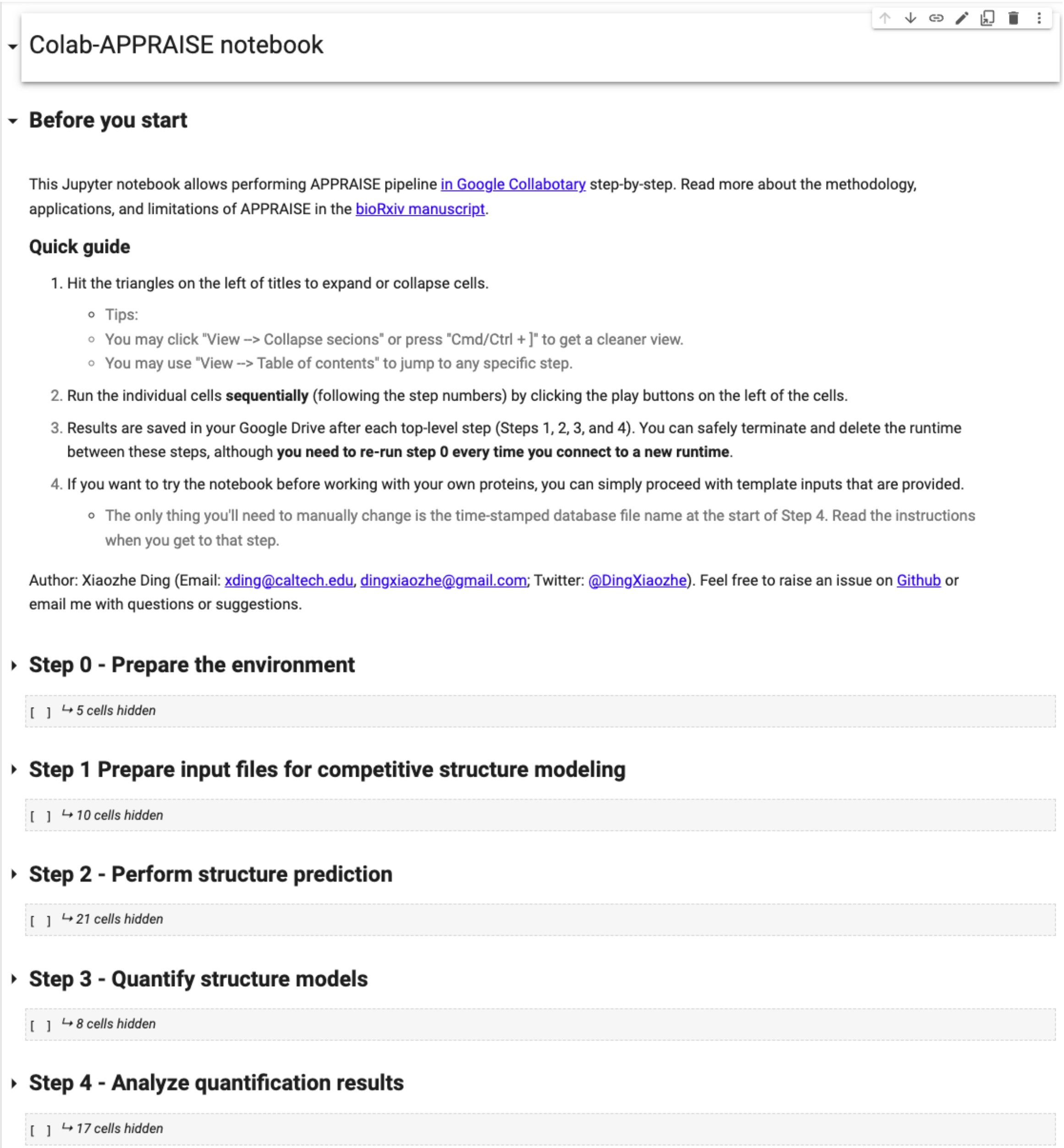

Figure S8 A screenshot of the interface of Colab-APPRAISE. APPRAISE can be easily accessed by running a web-based notebook on Google Colaboratory (https://tiny.cc/APPRAISE).

Table S1 Target receptor sequences and parameters used for APPRAISE analysis (part 1 of 2)

Receptor name	Organism	Uniprot accession ID	Domain used for modeling	Residue indices (start-end)	D_{max} (Å)	Axial ratio	R_{minor} (Å)	Anchor site	Sequence used for modeling
LY6A	Mus musculus (Mouse)	P05533	Mature protein	27-110	46.68	1.74	13.4	C-term	LECYQCYGVPFETSCPSITCPYPDGVCVTQEAAVIVDSQ<br>TRKVKNNLCLPICPPNIESMEILGTKVNVKTSCCQEDLC<br>NVAVP
PD-L1	Homo sapiens (Human)	Q9NZQ7	V domain	18-132	46.50	1.51	15.4	C-term	AFTVTVPKDLYVVEYGSNMTIECKFPVEKQLDLAALIVY<br>WEMEDKNIIQFVHGEEDLKVQHSSYRQRARLLKDQLSLG<br>NAALQITDVKLQDAGVYRCMISYGGADYKRITVKVNA
Beta-2 adrenergic receptor	Homo sapiens (Human)	P07550	TM1-TM7	29-342	93.8	2.23	21.0	N-term	DEVWVVGMGIVMSLIVLAIVFGNVLVITAIAKFERLQTV<br>TNYFITSLACADLVMGLAVVPFGAAHILMKMWTFGNFWC<br>EFWTSIDVLCVTASIETLCVIAVDRYFAITSPFKYQSLL<br>TKNKARVIILMVWIVSGLTSFLPIQMHWYRATHQEAINC<br>YANETCCDFFTNQAYAIASSIVSFYVPLVIMVFVYSRVF<br>QEAKRQLQKIDKSEGRFHVQNLSQVEQDGRTGHGLRRSS<br>KFCLKEHKALKTLGIIMGTFTLCWLPFFIVNIVHVIQDN<br>LIRKEVYILLNWIGYVNSGFNPLIYCRSPDFRIAFQELL<br>CL
Transferrin receptor 1	Homo sapiens (Human)	P02786	Ectodomain	122-760	86.50	1.47	29.4	N-term	LYWDDLKRKLSEKLDSTDFTGTIKLLNENSYVPREAGSQ<br>KDENLALYVENQFREFKLSKVWRDQHFVKIQVKDSAQNS<br>VIIVDKNGRLVYLVENPGGYVAYSKAATVTGKLVHANFG<br>TKKDFEDLYTPVNGSIVIVRAGKITFAEKVANAESLNAI<br>GVLIYMDQTKFPIVNAELSFFGHAHLGTGDPYTPGFPSF<br>NHTQFPPSRSSGLPNIPVQTISRAAAEKLFGNMEGDCPS<br>DWKTDSTCRMVTSESKNVKLTVSNVLKEIKILNIFGVIK<br>GFVEPDHYVVVGAQRDAWGPGAAKSGVGTALLLKLAQMF<br>SDMVLKDGFQPSRSIIFASWSAGDFGSVGATEWLEGYLS<br>SLHLKAFTYINLDKAVLGTSNFKVSASPLLYTLIEKTMQ<br>NVKHPVTGQFLYQDSNWASKVEKLTLDNAAFPFLAYSGI<br>PAVSFCFCEDTDYPYLGTTMDTYKELIERIPELNKVARA<br>AAEVAGQFVIKLTHDVELNLDYERYNSQLLSFVRDLNQY<br>RADIKEMGLSLQWLYSARGDFFRATSRLTTDFGNAEKTD<br>RFVMKKLNDRVMRVEYHFLSPYVSPKESPFRHVFWGSGS<br>HTLPALLENLKLRKQNNGAFNETLFRNQLALATWTIQGA<br>ANALSGDVWDIDNEF
Spike	SARS-CoV-2	P0DTC2	RBD	331-529	67.93	1.87	18.2	C-term	NITNLCPFGEVFNATRFASVYAWNRKRISNCVADYSVLY<br>NSASFSTFKCYGVSPTKLNDLCFTNVYADSFVIRGDEVR<br>QIAPGQTGKIADYNYKLPDDFTGCVIAWNSNNLDSKVGG<br>NYNYLYRLFRKSNLKPFERDISTEIYQAGSTPCNGVEGF<br>NCYFPLQSYGFQPTNGVGYQPYRVVVLSFELLHAPATVC<br>GPKK

Table S2 Target receptor sequences and parameters used for APPRAISE analysis (part 2 of 2)

Receptor name	PDB ID	Chain ID	Regions used for modeling	Construct length	D_{max} (Å)	Axial ratio	R_{minor} (Å)	Anchor site	Sequence used for modeling
FGFR2	1DJS	A	Structured region	216	105.22	3.06	17.19	C-term	TLEPEGAPYWTNTEKMEKRLHAVPAANTVKFRCPAGGNP<br>MPTMRWLKNGKEFKQEHRIGGYKVRNQHWSLIMESVVPS<br>DKGNYTCVVENEYGSINHTYHLDVVERSPHRPILQAGLP<br>ANASTVVGGDVEFVCKVYSDAQPHIQWIKHVEKNGSKYG<br>PDGLPYLKVLKAAGVNTTDKEIEVLYIRNVTFEDAGEYT<br>CLAGNSIGISFHSAWLTVLPA
TGF-β1	3KFD	A	Structured region	112	65.88	2.4	13.73	C-term	ALDTNYCFSSTEKNCCVRQLYIDFRKDLGWKWIHEPKGY<br>HANFCLGPCPYIWSLDTQYSKVLALYNQHNPGASAAPCC<br>VPQALEPLPIVYYVGRKPKVEQLSNMIVRSCKCS
CD3δ	1XIW	B	Structured region	74	48.7	2.06	11.82	C-term	MKIPIEELEDRVFVNCNTSITWVEGTVGTLLSDITRLDL<br>GKRILDPRGIYRCNGTDIYKDKESTVQVHYRMCQS
VirB8	4O3V	A	Cytoplasmic domain	143	56.27	1.62	17.37	C-term	ANPYISVANIMLQNYVKQREKYNYDTLKEQFTFIKNAST<br>SIVYMQFANFMNIDNSLSPVIRYQKLYRRSINIISINNI<br>NNNEATVTFESLAQNNTGEILENMLWEAKIGFIMDSIST<br>STLHNMPFHFIVTSYKLKLLRNKNQQ
PDGFR	3MJG	C	Ig-like C2-type domains 2&3	202	86.04	2.33	18.46	C-term	DERKRLYIFVPDPTVGFLPNDAEELFIFLTEITEITIPC<br>RVTDPQLVVTLHEKKGDVALPVPYDHQRGFSGIFEDRSY<br>ICKTTIGDREVDSDAYYVYRLQVSSINVSVNAVQTVVRQ<br>GENITLMCIVIGNEVVNFEWTYPRKESGRLVEPVTDFLL<br>DMPYHIRSILHIPSAELEDSGTYTCNVTESVNDHQDEKA<br>INITVVE
TrkA	2IFG	A	Ig-like C2-type domain 2	100	50.52	1.8	14.03	C-term	SFPASVQLHTAVEMHHWCIPFSVDGQPAPSLRWLFNGSV<br>LNETSFIFTEFLEPAANETVRHGCLRLNQPTHVNNGNYT<br>LLAANPFGQASASIMAAFMDNP
IL-7Rα	3DI3	B	Structured region	193	70.65	2.07	17.07	C-term	DYSFSCYSQLEVNGSQHSLTCAFEDPDVNTTNLEFEICG<br>ALVEVKCLNFRKLQEIYFIETKKFLLIGKSNICVKVGEK<br>SLTCKKIDLTTIVKPEAPFDLSVVYREGANDFVVTFNTS<br>HLQKKYVKVLMHDVAYRQEKDENKWTHVNLSSTKLTLLQ<br>RKLQPAAMYEIKVRSIPDHYFKGFWSEWSPSYYFRTP

Table S3 Sequences of engineered proteins used in APPRAISE tests in Figure 3

Protein Name	Description	Source	Protein sequence
12aa-B	Peptide from a phage display selection for Transferrin binding (positive)	Lee et al. 2001	THRPPMWSPVWP
12aa-B-scramble	Scrambled sequence of 12aa-B	Lee et al. 2001	PWRPSHPVWMPT
12aa-NB1	Peptide from a phage display selection for Transferrin binding (negative)	Lee et al. 2001	SSHMENTPDTLR
12aa-NB2	Peptide from a phage display selection for Transferrin binding (negative)	Lee et al. 2001	SNIRLSNSPMNT
12aa-NB3	Peptide from a phage display selection for Transferrin binding (negative)	Lee et al. 2001	YSYTPHATSMYS
12aa-NB4	Peptide from a phage display selection for Transferrin binding (negative)	Lee et al. 2001	SDMYPSTTLPVI
MNPD-1	Peptide rationally designed to bind to PD-L1	Yin et al. 2021	AQIREYKRCGQDEERVRRECKERGERQNCVYNIYKEGNCYVCGIICL
MOPD-1	Peptide rationally designed to bind to PD-L1	Yin et al. 2021	AQIREYKRCGQDEERVRRECKERGERQNCVYNIYKEGNCYVCGIICL
Scaffold	Scaffold of the designed peptides	Yin et al. 2021	GSEERRYKRCGQDEERVRRECKERGERQNCQYQIRKEGNCYVCEIRC
AAV9_560-606	Negative control sequence for PD-L1 binding (from AAV capsid)	negative control	ITNEEEIKTTNPVATESYGQVATNHQSAQAQAQTGWVQNQGILPGMV
PHP.eB_560-606	Negative control sequence for PD-L1 binding (from AAV capsid)	negative control	ITNEEEIKTTNPVATESYGQVATNHQSDGTLAVPFKAQAQTGWVQNQGILPGMV
AHB1	Miniproteins designed to bind to SARS-Cov-2-S	Cao et al. 2020	DEDLEELERLYRKAEEVAKEAKDASRRGDDERAKEQMERAMRLFDQVFELAQELQEKQTDGNRQKA THLDKAVKEAADELYQRVR
AHB2	Miniproteins designed to bind to SARS-Cov-2-S	Cao et al. 2020	ELEEQVMHVLDQVSELAHELLHKLTGEELERAAYFNWWATEMMLELIKSDDEREIREIEEEARRIL EHLEELARK
Influenza	Miniproteins designed to bind to Influenza virus	Chevalier et al., 2017	CIEQSFTTLFACQTAAEIWRAFGYTVKIMVDNGNCRLHVC
LCB1	Miniproteins designed to bind to SARS-Cov-2-S	Cao et al. 2020	DKEWILQKIYEIMRLLDELGHAEASMRVSDLIYEFMKKGDERLLEEAERLLEEVER
LCB2	Miniproteins designed to bind to SARS-Cov-2-S	Cao et al. 2020	SDDEDSVRYLLYMAELRYEQGNPEKAKKILEMAEFIAKRNNNEELERLVREVKKRL
LCB3	Miniproteins designed to bind to SARS-Cov-2-S	Cao et al. 2020	NDDELHMLMTDLVYEALHFAKDEEIKKRVFQLFELADKAYKNNDRQKLEKVVEELKELLERLLS
LCB4	Miniproteins designed to bind to SARS-Cov-2-S	Cao et al. 2020	QREKRLKQLEMLLEYAIERNDPYLMFDVAVEMLRLAEENNDERIIERAKRILEEYE
LCB5	Miniproteins designed to bind to SARS-Cov-2-S	Cao et al. 2020	SLEELKEQVKELKKELSPEMRRLIEEALRFLEEGNPAMAMMVLSDLVYQLGDPRVIDLYMLVTKT
AAV9_560-606	Negative control sequence for SARS-Cov-2-S binding (from AAV capsid)	Cao et al. 2020	ITNEEEIKTTNPVATESYGQVATNHQSAQAQAQTGWVQNQGILPGMV
PHP.eB_560-606	Negative control sequence for SARS-Cov-2-S binding (from AAV capsid)	Cao et al. 2020	ITNEEEIKTTNPVATESYGQVATNHQSDGTLAVPFKAQAQTGWVQNQGILPGMV
Nb80	Nanobody that binds to beta2 adrenergic receptor (parent)	Ring et al. 2013	QVQLQESGGGLVQAGGSLRLSCAASGSIFSINTMGWYRQAPGKQRELVAAIHSGGSTNYANSVKGR FTISRDNAANTVYLQMNSLKPEDTAVYYCNVKDYGAVLYEYDYWGQGTQVTVSS
Nb6A10	Nanobody selected to bind to beta2 adrenergic receptor using yeast display	Ring et al. 2013	QVQLQESGGGLVQAGGSLRLSCAASGGIFGFNTMGWYRQAPGKQRELVAAILSGGTTYYANSVKGR FTISRDNAANTVYLQMNSLKPEDTAVYYCNVKDHGSIIYDYDYWGQGTQVTVSS
Nb6B11	Nanobody selected to bind to beta2 adrenergic receptor using yeast display	Ring et al. 2013	QVQLQESGGGLVQAGGSLRLSCAASGTIFSINTMGWYRQAPGKQRELVAAIHSGGSTYYANSVKGR FTISRDNAANTVYLQMNSLKPEDTAVYYCNVKDYGAVLYDYDYWGQGTQVTVSS
Nb6A5	Nanobody selected to bind to beta2 adrenergic receptor using yeast display	Ring et al. 2013	QVQLQESGGGLVQAGGSLRLSCAASGSIFTFNTMGWYRQAPGKQRELVAAIHSGGNTDYANSVKGR FTISRDNAANTVYLQMNSLKPEDTAVYYCNVKDYGAIIYEYDYWGQGTQVTVSS
Nb6A9	Nanobody selected to bind to beta2 adrenergic receptor using yeast display	Ring et al. 2013	QVQLQESGGGLVQAGGSLRLSCAASGSIFAINTMGWYRQAPGKQRELVAAIHSGGSTNYANSVKGR FTISRDNAANTVYLQMNSLKPEDTAVYYCNVKDYGAIVYEYDYWGQGTQVTVSS
Nb6B9	Nanobody selected to bind to beta2 adrenergic receptor using yeast display	Ring et al. 2013	QVQLQESGGGLVQAGGSLRLSCAASGSIFALNIMGWYRQAPGKQRELVAAIHSGGTTNYANSVKGR FTISRDNAANTVYLQMNSLKPEDTAVYYCNVKDFGAIIYDYDYWGQGTQVTVSS
FGFR2_mb	Miniprotein designed to bind to FGFR2	Cao et al. 2022	DRRKEMDKVYRTAFKRITSTPDKEKRKEVVKEATEQLRRIAKDEEEKKKAAYMILFLKTLG
TGF-β1_mb	Miniprotein designed to bind to TGF-β1	Cao et al. 2022	HCTIEVVGVDPEKVEAIAAAYGAEVCEKDGKFEIHLDDPHSAESAAVAISVLTNRPVRLQC
CD3δ_mb	Miniprotein designed to bind to CD3δ	Cao et al. 2022	NHIACEIHNPEAAKEIAKVANVRRVYFIKQPGNRYFVLLKNADPEGVKKVRSKYNVRCVIRE
VirB8_mb	Miniprotein designed to bind to VirB8	Cao et al. 2022	NAEEITEKATLVGIEAWLLAKDEEQKKKVRTLNRQVKKLLQQNDLDQAKRVLDQLKSVLEDLKS
PDGFR_mb	Miniprotein designed to bind to PDGFR	Cao et al. 2022	DDERLATLAFRALIKRAGVKNLDVKVTNGKVRVTITGRDQASFKALQLVFALARLGLQVQIDTR
TrkA_mb	Miniprotein designed to bind to TrkA	Cao et al. 2022	RDEIKERIFKAVVRAIVTGNPEQLKEAKKLLEKLKKLGRLDQDAKKFEKAIRQVEKRLRS
IL-7Rα_mb	Miniprotein designed to bind to IL-7Rα	Cao et al. 2022	SVIEKLRKLEKQARKQGDEVLVMLARMVLEYLEKGWVSEEDADESADRIEEVLKK
LCB1	Miniproteins designed to bind to SARS-Cov-2-S	Cao et al. 2020	DKEWILQKIYEIMRLLDELGHAEASMRVSDLIYEFMKKGDERLLEEAERLLEEVER

Table S4 Peptides used for *in silico* screening

Peptide name	Peptide sequence (residues 587-594 in VP1)	Original source
AAV9	AQ-------AQAQTG	Gao et al., 2002
PHP.B	AQTLAVPFKAQAQTG	Deverman et al. 2016
PHP.D	AQWKNMGLQAQAQTG	Unpublished in vivo selection
SRK-1	AQLYHGGSTAQAQTG	Kumar et al. 2020
SRK-2	AQNNSVRQLAQAQTG	Kumar et al. 2020
SRK-3	AQVNSTRNVAQAQTG	Kumar et al. 2020
SRK-4	AQGNMTKFTAQAQTG	Kumar et al. 2020
SRK-5	AQTAIQPPKAQAQTG	Kumar et al. 2020
SRK-6	AQITTDQPFAQAQTG	Kumar et al. 2020
SRK-7	AQDTANTARAQAQTG	Kumar et al. 2020
SRK-8	AQTHDAQAWAQAQTG	Kumar et al. 2020
SRK-9	AQQPLAEEAAQAQTG	Kumar et al. 2020
SRK-10	AQTALANQKAQAQTG	Kumar et al. 2020
SRK-11	AQTGTERLSAQAQTG	Kumar et al. 2020
SRK-12	AQNGVTQSKAQAQTG	Kumar et al. 2020
SRK-13	AQWTEQRLVAQAQTG	Kumar et al. 2020
SRK-14	AQDTGLNNRAQAQTG	Kumar et al. 2020
SRK-15	AQPLPPTSIAQAQTG	Kumar et al. 2020
SRK-16	AQSDPGKFMAQAQTG	Kumar et al. 2020
SRK-17	AQTTMGTMLAQAQTG	Kumar et al. 2020
SRK-18	AQKQTQDSSAQAQTG	Kumar et al. 2020
SRK-19	AQLAHNSALAQAQTG	Kumar et al. 2020
SRK-20	AQVVPSTYRAQAQTG	Kumar et al. 2020
SRK-21	AQFRHLTGAAQAQTG	Kumar et al. 2020
SRK-22	AQSANLLSSAQAQTG	Kumar et al. 2020
SRK-23	AQFSNTHALAQAQTG	Kumar et al. 2020
SRK-24	AQFNSKLQLAQAQTG	Kumar et al. 2020
SRK-25	AQFKTNISAAQAQTG	Kumar et al. 2020
SRK-26	AQYPVPLKQAQAQTG	Kumar et al. 2020
SRK-27	AQHVNHMAPAQAQTG	Kumar et al. 2020
SRK-28	AQIVSNQMSAQAQTG	Kumar et al. 2020
SRK-29	AQPRPERMYAQAQTG	Kumar et al. 2020
SRK-30	AQNMKIQHVAQAQTG	Kumar et al. 2020
SRK-31	AQNTNVPAMAQAQTG	Kumar et al. 2020
SRK-32	AQSAQLRSSAQAQTG	Kumar et al. 2020
SRK-33	AQSHHEQVSAQAQTG	Kumar et al. 2020
SRK-34	AQGATGHLTAQAQTG	Kumar et al. 2020
SRK-35	AQHNLRDSIAQAQTG	Kumar et al. 2020
SRK-36	AQGPGTSFKAQAQTG	Kumar et al. 2020
SRK-37	AQSPPVQGLAQAQTG	Kumar et al. 2020
SRK-38	AQTLYNAIHAQAQTG	Kumar et al. 2020
SRK-39	AQLGDITGFAQAQTG	Kumar et al. 2020
SRK-40	AQGFNSMKPAQAQTG	Kumar et al. 2020
SRK-41	AQSNGLNGLAQAQTG	Kumar et al. 2020
SRK-42	AQVRIPGALAQAQTG	Kumar et al. 2020
SRK-43	AQDMGTDNLAQAQTG	Kumar et al. 2020
SRK-44	AQNYATKSQAQAQTG	Kumar et al. 2020
SRK-45	AQSVTTSHVAQAQTG	Kumar et al. 2020
SRK-46	AQTSGTDGIAQAQTG	Kumar et al. 2020
SRK-47	AQARTAHGYAQAQTG	Kumar et al. 2020
SRK-48	AQHSANMSKAQAQTG	Kumar et al. 2020
SRK-49	AQHDERANMAQAQTG	Kumar et al. 2020
SRK-50	AQNNFNASLAQAQTG	Kumar et al. 2020
SRK-51	AQSASLVSHAQAQTG	Kumar et al. 2020
SRK-52	AQAPRIDNAAQAQTG	Kumar et al. 2020
SRK-53	AQLTSSNALAQAQTG	Kumar et al. 2020
SRK-54	AQTLNSIRAAQAQTG	Kumar et al. 2020
SRK-55	AQSGTGRQQAQAQTG	Kumar et al. 2020
SRK-56	AQKTTLASGAQAQTG	Kumar et al. 2020
SRK-57	AQMRVNTEEAQAQTG	Kumar et al. 2020
SRK-58	AQFETLHKTAQAQTG	Kumar et al. 2020
SRK-59	AQTQHRFEMAQAQTG	Kumar et al. 2020
SRK-60	AQHTAEKAPAQAQTG	Kumar et al. 2020
SRK-61	AQNHMVRELAQAQTG	Kumar et al. 2020
SRK-62	AQRFQPSSAAQAQTG	Kumar et al. 2020
SRK-63	AQRSVANVPAQAQTG	Kumar et al. 2020
SRK-64	AQVFQATRTAQAQTG	Kumar et al. 2020
SRK-65	AQEQRTPSPAQAQTG	Kumar et al. 2020
SRK-66	AQGSSTASLAQAQTG	Kumar et al. 2020
SRK-67	AQQVPHLHSAQAQTG	Kumar et al. 2020
SRK-68	AQPSQPYTKAQAQTG	Kumar et al. 2020
SRK-69	AQTHTRDQGAQAQTG	Kumar et al. 2020
SRK-70	AQINPGITLAQAQTG	Kumar et al. 2020
SRK-71	AQLQPTKSSAQAQTG	Kumar et al. 2020
SRK-72	AQQDAKVTTAQAQTG	Kumar et al. 2020
SRK-73	AQGASTHNAAQAQTG	Kumar et al. 2020
SRK-74	AQIPVSIQAAQAQTG	Kumar et al. 2020
SRK-75	AQVTSAHPVAQAQTG	Kumar et al. 2020
SRK-76	AQTASLIASAQAQTG	Kumar et al. 2020
SRK-77	AQDRGTRTVAQAQTG	Kumar et al. 2020
SRK-78	AQTAYLEVKAQAQTG	Kumar et al. 2020
SRK-79	AQATTQMSSAQAQTG	Kumar et al. 2020
SRK-80	AQKYDASQSAQAQTG	Kumar et al. 2020
SRK-81	AQTGTSHLHAQAQTG	Kumar et al. 2020
SRK-82	AQTMTPSGIAQAQTG	Kumar et al. 2020
SRK-83	AQTPSSSGNAQAQTG	Kumar et al. 2020
SRK-84	AQKDVVNSNAQAQTG	Kumar et al. 2020
SRK-85	AQRSPATMLAQAQTG	Kumar et al. 2020
SRK-86	AQYDQKSLAAQAQTG	Kumar et al. 2020
SRK-87	AQMGARNLPAQAQTG	Kumar et al. 2020
SRK-88	AQLPISATEAQAQTG	Kumar et al. 2020
SRK-89	AQTRHTSLTAQAQTG	Kumar et al. 2020
SRK-90	AQNKLTANGAQAQTG	Kumar et al. 2020
SRK-91	AQNGDSHSHAQAQTG	Kumar et al. 2020
SRK-92	AQVRTDMDMAQAQTG	Kumar et al. 2020
SRK-93	AQSVSTPRGAQAQTG	Kumar et al. 2020
SRK-94	AQVSRQFEPAQAQTG	Kumar et al. 2020
SRK-95	AQSANNVRGAQAQTG	Kumar et al. 2020
SRK-96	AQIGTKSTNAQAQTG	Kumar et al. 2020
SRK-97	AQGSELRTGAQAQTG	Kumar et al. 2020
